# Murine Q Fever Vaccination Model Reveals Sex Dimorphism in Early Phase Delayed-Type Hypersensitivity Responses

**DOI:** 10.3389/fimmu.2022.894536

**Published:** 2022-06-15

**Authors:** Picabo Binette, Mahelat Tesfamariam, Diane Cockrell, Robert A. Heinzen, Crystal Richards, Carl Shaia, Carrie Mae Long

**Affiliations:** ^1^ Laboratory of Bacteriology, Intramural Research Program, National Institute of Allergy and Infectious Disease, National Institutes of Health, Hamilton, MT, United States; ^2^ Rocky Mountain Veterinary Branch, Intramural Research Program, National Institute of Allergy and Infectious Disease, National Institutes of Health, Hamilton, MT, United States

**Keywords:** *Coxiella burnetii*, DTH (delayed type hypersensitivity), vaccine allergy, sex dimorphism, Q fever Vaccine (Q-VAX^®^), bacterial vaccine

## Abstract

Delayed-type hypersensitivity (DTH) responses to microbial vaccines and related components are a major roadblock for widespread licensing of whole cell vaccines such as that of Q fever. Q fever is a zoonotic disease caused by the intracellular bacterium *Coxiella burnetii*. The only currently licensed vaccine, Q-Vax^®^, is a whole cell inactivated formulation that is associated with a potentially severe dermal post vaccination DTH response in previously sensitized individuals. To investigate the underlying immunologic mechanisms of this response and better represent the early-phase DTH response observed in humans, a murine sensitization and skin testing model was developed and employed. Female C57Bl/6J mice displayed the most robust early-phase DTH responses following sensitization and elicitation compared to their male counterparts and other mouse strains. Immunologic responses were measured within the skin, draining lymph nodes, and serum following both sensitization and elicitation with Q fever whole cell vaccines. Local immunologic responses in the dermis were characterized by inflammation primarily involving neutrophils, macrophages, and T cells. Secondary lymphoid organ profiling revealed distinct immunological signatures following both sensitization and elicitation with a sex-based dichotomy in T cell phenotypes and antigen presenting cell numbers. Beyond providing a post-Q fever vaccination DTH model that recapitulates early-phase DTH events, these data suggest that sex is a primary factor influencing the magnitude and composition of the ensuing response.

## Introduction

Delayed-type hypersensitivity (DTH) response in the skin is characterized by an antigen-specific allergic reaction leading to erythema and induration at the site of antigen exposure following sensitization and elicitation. DTH is distinguished from other forms of hypersensitivity based on the delayed appearance of pathology compared to the more immediate responses of other hypersensitivity reactions. This is due to the underlying immunologic factors of each form of hypersensitivity; immediate types are antibody mediated while DTH is generally T cell mediated. Traditionally, DTH has been subdivided into several major subtypes, including: contact, granulomatous, Jones-Mote/cutaneous basophil hypersensitivity, and tuberculin reactions (1). An updated classification scheme divides DTH reactions into four major types (I-IV) based on the primary effector cells and dominant T cell phenotype involved (2, 3). Despite exhibiting distinct immunological and pathological features, these reactions generally involve an initial influx of immune cells (e.g. basophils, macrophages, and neutrophils) at the site of antigen exposure followed by antigen-specific T cells by 48 hours (1) in sensitized individuals. The immunologic makeup of the cellular infiltrate is dependent on multiple factors, including the nature of the offending antigen and the species experiencing the reaction.

Vaccine-induced DTH reactions are relatively rare among currently licensed vaccines with reported frequencies of 0.35-1.18% (4). Aluminum is a major allergenic culprit among vaccine ingredients, typically causing contact dermatitis and subcutaneous granulomas in children receiving aluminum-adsorbed vaccines (5). In addition to aluminum, whole cell vaccines (WCV) and their microbial derivatives can cause post-vaccination DTH events such as those caused by *Coxiella burnetii* WCV. *C. burnetii* is gram-negative bacterium and the causative agent of the human disease Q fever. This disease spans a range of clinical presentations including acute Q fever and persistent focalized infections (also known as chronic Q fever) and may lead to sequelae such as post-acute Q fever chronic fatigue syndrome (6). Although human infection with *C. burnetii* typically occurs due to contact with livestock or infectious byproducts (7), this pathogen also poses a risk of misuse *via* bioterrorism (8). A formalin-inactivated WCV, Q-Vax^®^ (Seqirus^™^), is licensed in Australia for the prevention of Q fever in at-risk occupational groups. This vaccine is remarkably effective at inducing durable, protective immune responses (9); however, its widespread deployment is precluded by the potential for a severe post-vaccination DTH response in pre-sensitized individuals (10). Indeed, Q-Vax^®^ administration is marked by cumbersome pre-vaccination requirements including serologic and skin testing (11). Elimination of the post-vaccination DTH response is a desirable, if not obligatory, characteristic of a next-generation, widely deployable Q fever vaccine (12).

Although human Q fever vaccine-associated DTH reactions have not been well-characterized immunologically, clinical observations suggest a biphasic DTH response (13), possibly involving tuberculin-type and granulomatous DTH characterized by two peaks of skin reactivity (days 1-2 and 9) (14). Currently, guinea pig models are the most well-developed (15), with animals displaying delayed erythema and induration kinetics (16) along with abscess and granuloma formation (17). Importantly, quantifiable erythema and induration typically occur no earlier than 7 days following skin testing in guinea pig models, suggesting that the ensuing response is primarily that of late-phase or granulomatous DTH. Indeed, Ascher, et al. suggest that the guinea pig model shows “pure late reactivity without significant DTH” (18), referring to granulomatous DTH as “late reactivity”. Beyond T cell-mediated DTH responses, the role of B cells, antibody, and antigen-antibody complexes in granuloma formation and persistence should be considered given the high *C. burnetii*-specific complement fixation antibody titers documented in human sterile abscess fluid (14, 19). Although the guinea pig is an excellent physiologic model of both *C. burnetii* infection (20) and post-vaccination granulomatous DTH (21), its utility is limited by a paucity of species-specific experimental tools. To expand the framework for understanding the mechanisms underlying the post-vaccination DTH response, murine models have been proposed. In 1987, Kazar, et al. introduced a model involving a 100 µg intraperitoneal sensitization dose followed by WCV footpad injection (22). Recently, Fratzke, et al. introduced a murine model involving subcutaneous WCV sensitization (50 µg) followed by a 5-6 week resting period and subcutaneous WCV elicitation with a variety of WCV doses in SKH-1 and C57Bl/6J mice (23). This study revealed that Q fever post-vaccination DTH was characterized by a robust local Th1 DTH response and necrotic lesions at 14 days post skin testing, likely similar to that of the late-phase of the human condition (19). While this component of the response is clearly important, the early phase DTH response may translate more readily to that of pre-immune humans, who experience measurable dermal reactions prior to 7 days following skin testing when erythema diameter is measured (13). Indeed, human skin tests can be read as early as 24-48 hours post injection for accurate prediction of potential adverse post-vaccination DTH responses (14, 22, 24) and a 4 day waiting period following a WCV “test” administration was considered adequate to predict adverse DTH responses (25). While granulomatous DTH reactions are likely responsible for severe adverse post-Q fever vaccination dermal sequelae given the persistent nature of WCV in the skin (14), these reactions are preceded by significant, measurable early phase DTH responses in humans (13, 26). Therefore, recapitulation of the early phase DTH response following Q fever vaccination is an important consideration in animal model development that has not yet been addressed. Additionally, these responses occur earlier than granulomatous responses, allowing for more rapid screening of vaccine candidates and analysis of initial immunologic events preceding later pathology.

Further insight is needed into the host immunologic mechanisms and vaccine-related factors driving the post-Q fever vaccination DTH response. Enhanced model development is a key component of this goal. The focus on granulomatous DTH responses and dearth of models representing the early phase response represent a major area of oversight. To address this, we developed a murine model of post-Q fever dermal reactogenicity which recapitulates the early phase DTH response observed in pre-immune humans following vaccination and/or skin testing. We evaluated various mouse strains and sensitization schema to develop an optimal model which displayed measurable early phase responses distinguishable from unsensitized, WCV skin tested controls. We then characterized local and systemic immune responses of female and male mice, revealing sex-based dimorphism. Models such as this are critical for the development of an improved Q fever vaccine and will yield important immunologic insight into vaccine related DTH responses.

## Methods

### Whole Cell Vaccines (WCV)

For WCV preparation, *C. burnetii* strains were grown in acidified citrate cysteine medium-2 (27) at 37 °C, 2.5% O_2_, and 5% CO_2_, fixed in 4% paraformaldehyde for at least 12 h, washed in sterile PBS, resuspended in USP-grade saline, and stored at −80 °C until use. WCV concentration was determined *via* direct bacterial count as previously described (16, 28). *C. burnetii* NMI Δ*dot/icm* was previously described (16). All manipulations of phase I *C. burnetii* stocks and infected animal tissue were performed in a BSL-3 laboratory in accordance with standard operating procedures approved by the Rocky Mountain Laboratories Institutional Biosafety Committee.

### Mice

BALB/cJ and C57Bl/6J mice were obtained from Jackson Laboratories and SKH1-Elite mice were obtained from Charles River at 6 to 10 weeks of age. Animals were acclimated for at least a week prior to experimental manipulation. Animals were housed in same-sex groups in individually ventilated plastic cages (Super Mouse 750™ Ventilated Cages, Lab Products LLC, Seaford, DE, USA) with autoclaved bedding (Sani-Chips^®^, P.J. Murphy Forest Product Corp., Montville, NJ, USA), and enrichment (ALPHA Nest^®^, Shepherd Specialty Papers, Watertown, TN, USA). Commercial rodent diet (2016 Teklad global 16% protein rodent diet, Envigo Teklad, Denver, CO, USA) and chlorinated, reverse osmosis filtered tap water were administered *ad libitum*. A 12-h light–dark cycle was maintained in animal housing facilities which were kept at 22°C +/- 2°C and 40–60% relative humidity with a 50% set point. For all experiments, group numbers equaled 5-9 animals with the exception of SKH1 mice in the single dose experiment (n=4), the female C57Bl/6J NMI : NMI group in the repeated sensitization experiment (n=8), and the male C57Bl/6J Saline : Saline group in the repeated sensitization experiment (n=7). Five mice were housed per cage apart from the aforementioned groups, which were housed from 1-4 mice per cage. All animals were housed in approved animal biosafety level 2 (ABSL-2) facilities and manipulated under ABSL-2 standard operating procedures approved by the Rocky Mountain Laboratories Institutional Biosafety Committee and an Institutional Animal Care and Use Committee-approved protocol (ASP-2020-032-E). Animal experiments and procedures were performed in an Association for Assessment and Accreditation of Laboratory Animal Care-accredited National Institute of Allergy and Infectious Diseases animal facility.

### Sensitization, Skin Testing, and Monitoring

**Figure 1:**
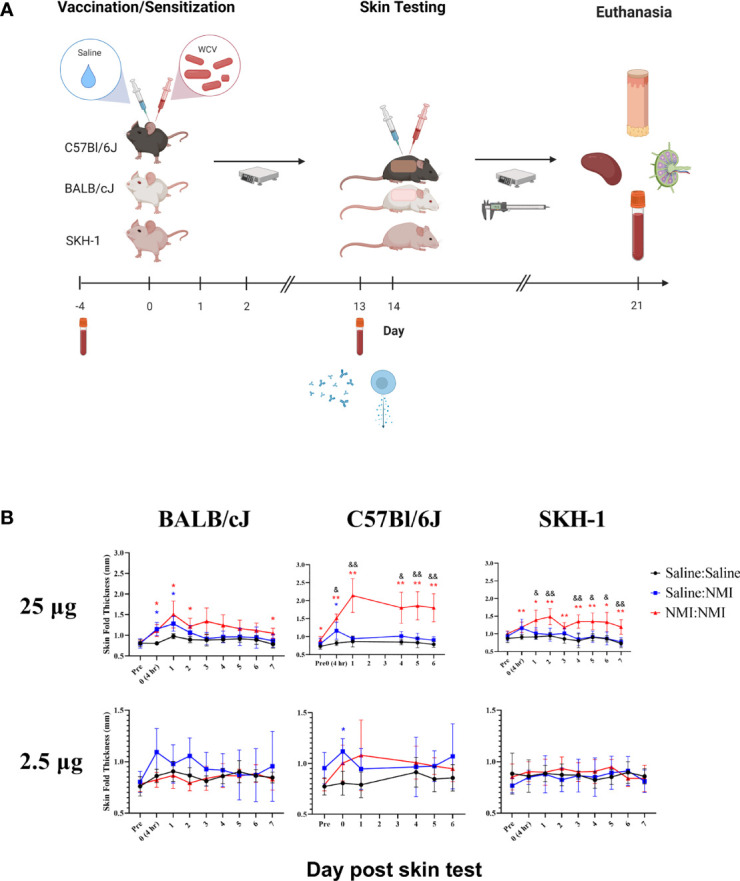
Repeated sensitization model yields robust early-phase DTH response in C57Bl/6 mice The murine repeated sensitization model is outlined in **(A)** Syringes indicate saline or WCV injections and tubes indicate blood collection. Skin thickness kinetics are displayed the repeated sensitization model **(B)**. *p ≤ 0.05, **p ≤ 0.01 compared to Saline : Saline control group means; ^&^ *p* ≤ 0.05, ^&&^ *p* ≤ 0.01 compared to Saline : NMI unsensitized control group means. Symbols correspond to subject group by color.

Mice were subcutaneously administered 25 µg WCV in USP-grade saline or USP-grade saline alone within a 0.5 mL volume in the right flank to induce sensitization. For the repeated sensitization protocol, this was performed three times daily. For the single sensitization studies (**Supplementary Figure 1A**), mice were skin tested 21 days following vaccination. For repeated sensitization studies (**Figure 1A**), mice were skin tested 14 days following initial vaccination. Skin tests were performed *via* intradermal injections at two sites on the back. 25 and 2.5 µg WCV in USP-grade saline or USP-grade saline alone were administered in 0.05 mL volumes. BALB/cJ and C57Bl/6J mice were shaved at injection sites immediately prior to skin testing. Non-terminal blood collections were performed *via* cheek bleed as indicated in **Figure 1A** and **Supplementary Figure 1A**. Inhalation sedation *via* isoflurane was employed during vaccination, blood collection, and skin testing procedures. Body weight, clinical scores, and skin metrics (e.g. erythema area, thickness) were assessed one day prior to and daily following skin testing. Skin thickness was measured using a digimatic micrometer (Mitutoyo 700-118-30) at each skin testing site. No measurable erythema was observed in any mice during the study.

### Euthanasia, Tissue Collection, and Processing

For all experiments, mice were humanely euthanized in accordance with the American Veterinary Medical Association's guidelines for the euthanasia of animals. Blood was collected by cardiac puncture. Following euthanasia, draining lymph nodes (25 µg site: brachial; 2.5 µg site: inguinal) were collected and placed into tubes containing sterile phosphate-buffered saline (PBS; Gibco, pH: 7.2, cat. n. 20012-D27). 10 mm skin biopsies (Acuderm P1025) were collected and cut in half using surgical scissors and forceps. One skin biopsy half was placed into 5 mL PBS for cellular processing and the other half was placed into 5 mL 10% neutral buffered formalin for histopathological processing.

Blood was spun at 1100 xg for 10 minutes and sera was collected and stored at -20°C for subsequent analysis. Lymph nodes were manually dissociated using the frosted ends of two microscope slides. To obtain single cell suspensions from skin samples in PBS, biopsy halves were processed as described by Kashem and Kaplan (29), with modifications. For epidermal separation, 1 mL/cm dispase solution (Dispase II, ThermoFisher, 17105041) was used for gentle separation. 6-well plates were used for epidermal separation and 24-well plates were used for dermal processing, with 1 biopsy half per well. Dermis and lymph node cellularity were determined using a Cellaca MX automated cell counter (Nexcelom) or Scepter automated cell counter (Millipore, PHCC20060) with size exclusion parameters (6 to 36 μm).

### Flow Cytometry

Single-cell suspensions from the dermis and lymph nodes were aliquoted into 96-well U-bottom plates at a minimum density of 10^6^ cells per well. Whole epidermal cell suspensions were plated individually and counted using precision count beads (BioLegend, 424902). Cells were resuspended in PBS and stained for viability using the Zombie Yellow™ Fixable Viability Kit (BioLegend, 423104) following manufacturer’s instructions. Cells were washed in PBS then in staining buffer (PBS + 1% bovine serum albumin). Next, cells were stained with a cocktail of antibodies specific for mouse cell surface antigens, as detailed in **Supplementary Table 1**. Following surface staining, cells were washed in staining buffer and fixed in Cytofix (BD, cat. n. 554655). Samples in panels requiring intracellular staining (Dermis and dLN 2) were fixed and intracellularly stained using the Foxp3 Transcription Factor Staining Buffer Set (eBioscience™, 00-5523-00) according to manufacturer’s instructions. Following fixation and/or intracellular staining, cells were washed in staining buffer and analyzed on a BD LSR II or FACSymphony flow cytometer using FacsDiva software (BD Biosciences). A minimum of 20,000 events were captured for each sample. Data analysis was performed with FlowJo 10.0 software (TreeStar Inc., Ashland, Oregon). Single-stained compensation controls and fluorescence minus one staining controls were included to help set gating boundaries. Gating strategies are displayed in **Supplementary Figure 2**.

### Cytokine Detection and Serology

Cytokines were detected using LEGENDplex™ assays (BioLegend) according to manufacturer’s protocols. For lymph node cytokine detection, the mouse T Helper Cytokine Panel Version 3 (741044) was utilized with undiluted single cell suspension in duplicate. For sera cytokine detection, the mouse Cytokine Panel 2 (740134) was utilized with mouse sera diluted 1:2 in duplicate.

*C. burnetii* phase I and II IgG and phase II IgM antibodies were qualitatively determined in mouse sera using GenWay *Coxiella burnetii* (Q-Fever) Phase I (GWB-FEA920) and Phase II (GWB-2EB8D1) IgG and Phase II IgM (GWB-F771C4) ELISA kits. Manufacturer’s protocols were followed, with several exceptions. Stored sera was thawed and diluted in sample diluent 1:8 and 1:10 for phase I and phase II ELISAs, respectively. 100 µL of diluted sera from each animal, was added to respective wells of supplied microtiter strip wells precoated with phase I/II antigens along with 100 µL of negative, positive, and cutoff controls supplied in the kit. All experimental samples were assayed in duplicate. For antibody detection, horseradish peroxidase (HRP) labeled anti-human IgG or IgM conjugate was added to control wells while a 1:5000 HRP anti-Mouse IgG antibody (Pierce 31430) or HRP anti-mouse IgM antibody (ThermoFisher 31440) diluted in sample eluent was added to wells containing mouse sera. Absorbance at 450nm (reference wavelength 600 nm) was measured for each sample using a GloMax^®^ (Promega™) microwell plate reader.

### Histology

Skin biopsies were fixed in 10% Neutral Buffered Formalin for 48 hours, placed in tissue cassettes and processed with a Sakura VIP-6 Tissue Tek on a 12 h automated schedule using a graded series of ethanol, xylene, and PureAffin. Embedded tissues were sectioned at 5 μm, mounted and dried overnight at 42°C prior to staining with hematoxylin and eosin using established methods. Biopsy specimens were evaluated using an Olympus BX53 microscope.

### Statistical Analysis

Statistical analyses were conducted using GraphPad Prism version 7.0 (GraphPad Software, La Jolla, CA, USA). Statistical evidence for differences in group means were assessed using two-sample Welch *t* tests, allowing for unequal variances between groups. For each comparison, we computed Wald-type 95% confidence intervals and describe statistical significance with two-sided *p*-values. Statistical evidence for differences in group means over time (e.g. skin fold thickness) were assessed using two-way ANOVAs or mixed-effects analysis, with no assumption of sphericity. For each comparison, we enlisted the Geisser-Greenhouse correction. No adjustment was made for multiple comparisons due to the small sample sizes involved. We represent *p*-values in equal to or below 0.05 with a single asterisk (*) or ampersand (&) and *p*-values equal to or below 0.01 with a double asterisk (**) or ampersand (&&) unless otherwise indicated. Error bars represent standard deviation of group mean.

## Results

### Repeated NMI WCV Sensitization Protocol Results in Early-Phase DTH Responses in C57Bl/6J and SKH-1 Mice

For initial model development, we evaluated DTH responses in three mouse strains (BALB/cJ, C57Bl/6J, and SKH-1). BALB/cJ and C57Bl/6J mice were selected based on their Th2/Th1 biases, respectively (30). SKH-1 mice were selected due to their hairless phenotype and intact immune system (31). In initial studies, female mice were sensitized with a single 25 µg dose of WCV or saline followed by a 20 day rest period. Mice were intradermally skin tested with 25 and 2.5 µg doses of WCV or saline 21 days following initial vaccination and monitored for 21 days following skin testing (**Supplementary Figure 1A**). During this period, mice exhibited neither weight loss (data not shown) nor changes in skin thickness (**Supplementary Figure 1B**). In a subsequent experiment, the sensitization potency was increased by repeating vaccination twice for a total of 3 daily injections (**Figure 1A**). Following a 13-day resting period (from the day of the initial vaccination), mice were intradermally skin tested as described above and monitored for a week. While mice did not display weight loss (data not shown), significant increases in skin thickness were observed at 25 µg testing sites in all strains at NMI : NMI sites compared to mock Saline : Saline and unsensitized Saline : NMI controls at various time points post skin testing. Skin thickness peaked in magnitude at days 1-2 post skin testing (**Figure 1B**). No significant increases in skin thickness were observed at 2.5 µg testing sites with the exception of day 0 C57Bl/6J values. The magnitude of skin thickness and ability to discern the variable magnitudes of unsensitized and sensitized responses was optimal for C57Bl/6J animals.

**Figure 2:**
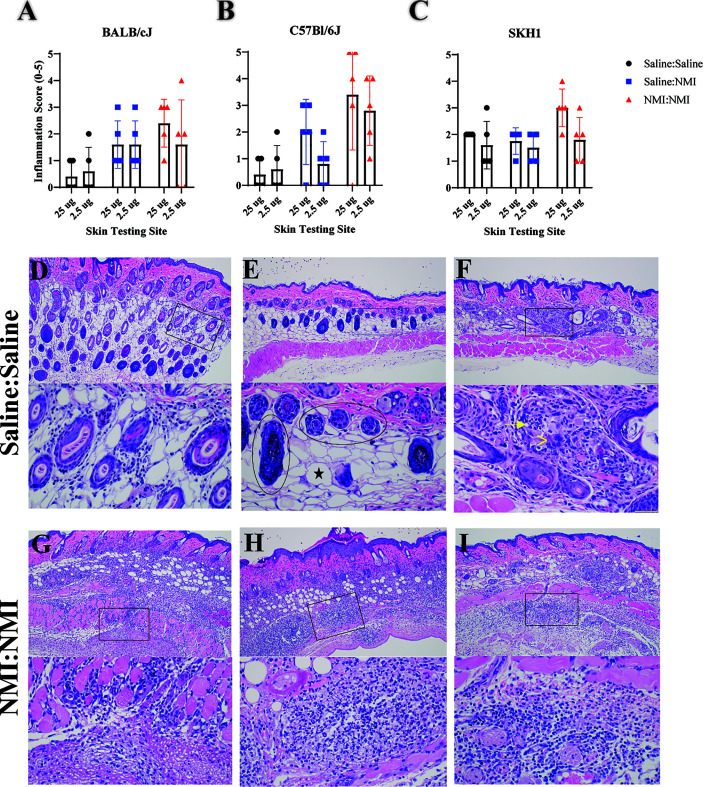
Histologic analysis of murine skin reveals WCV-associated inflammation. Qualitative inflammation scoring was performed on skin biopsies from BALB/cJ **(A)**, C57Bl/6J **(B)**, and SKH-1 **(C)** mice at 7 days post skin testing. Histologic skin pathology not associated with vaccination are depicted in **(D–F)**. Histologic skin lesions associated with vaccination are depicted in **(G–I)**. Top panels represent 100X magnification and lower panels represent 400X magnification. Magnified inset areas are demarcated by rectangles in top panels. In **(E)**, the circled areas denote hair follicles and the black star denotes an adipocyte. In **(F)**, the arrow denotes neutrophils and (>) denotess multinucleated giant cells.

Skin biopsy halves were processed at 7 days post skin testing and histopathological analysis was performed on hematoxylin and eosin (H&E)-stained tissue sections. An inflammation scoring scheme was developed (**Supplementary Figure 3**) and applied to sections from mice that had undergone repeated sensitization and skin testing (**Figure 1A**). Inflammation scores revealed a lack of discernable response between Saline : NMI and NMI : NMI groups in BALB/cJ mice (**Figure 2A**). In contrast, C57Bl/6J mice exhibited generally more robust inflammatory scores following NMI : NMI treatment at both skin testing doses compared to Saline : Saline and Saline : NMI treatments (**Figure 2B**). While SKH-1 NMI : NMI animals exhibited potentially increased inflammation at the 25 µg skin testing site, baseline inflammation scores of Saline : Saline animals served as a confounding factor for comparison (**Figure 2C**). Representative histological images from Saline : Saline or Saline : NMI (**Figures 2D–F**) and NMI : NMI (**Figures 2G–1**) reflect these scores. Saline control BALB/cJ mice displayed minimal multifocal inflammation likely associated with injection (**Figure 2D**). Inflammation among the hair follicles and adipose cells consisted primarily of neutrophils and macrophages. Saline control C57Bl/6J mice did not exhibit any visible inflammation in the epidermis, dermis, hypodermis, panniculus muscle or deep fascia (**Figure 2E**). Inflammation in sensitized mice was generally characterized by macrophages, lymphocytes, and neutrophils. BALB/cJ mice experienced inflammation from the dermis to fascia deep to the panniculus muscle, consisting of macrophages, lymphocytes, and occasional neutrophils (**Figure 2G**). Sensitized C57Bl/6J mice also experienced inflammation with multiple foci of intense inflammation with degenerate neutrophils and necrosis within the hypodermis, panniculus muscle, and deep fascia (**Figure 2H**). Sensitized SKH-1 mice exhibited inflammation similar to that of BALB/cJ mice (**Figure 2I**). Importantly, Saline : Saline control SKH-1 mice exhibited pyogranulomatous inflammation within the hypodermis consisting of neutrophils, macrophages and multinucleated giant cells surrounding keratin and fragments of hair follicles present as a complication of the hairless gene (**Figure 2F**), reducing the utility of this strain in our modeling system. Indeed, we chose to move forward with C57BL/6J mice as our strain of choice due to their robust early-phase DTH responses that were discernable from negative (Saline: Saline) and unsensitized (Saline: NMI) controls.

### Female and Male C57Bl/6J Mice Exhibit Divergent DTH Responses

**Figure 3:**
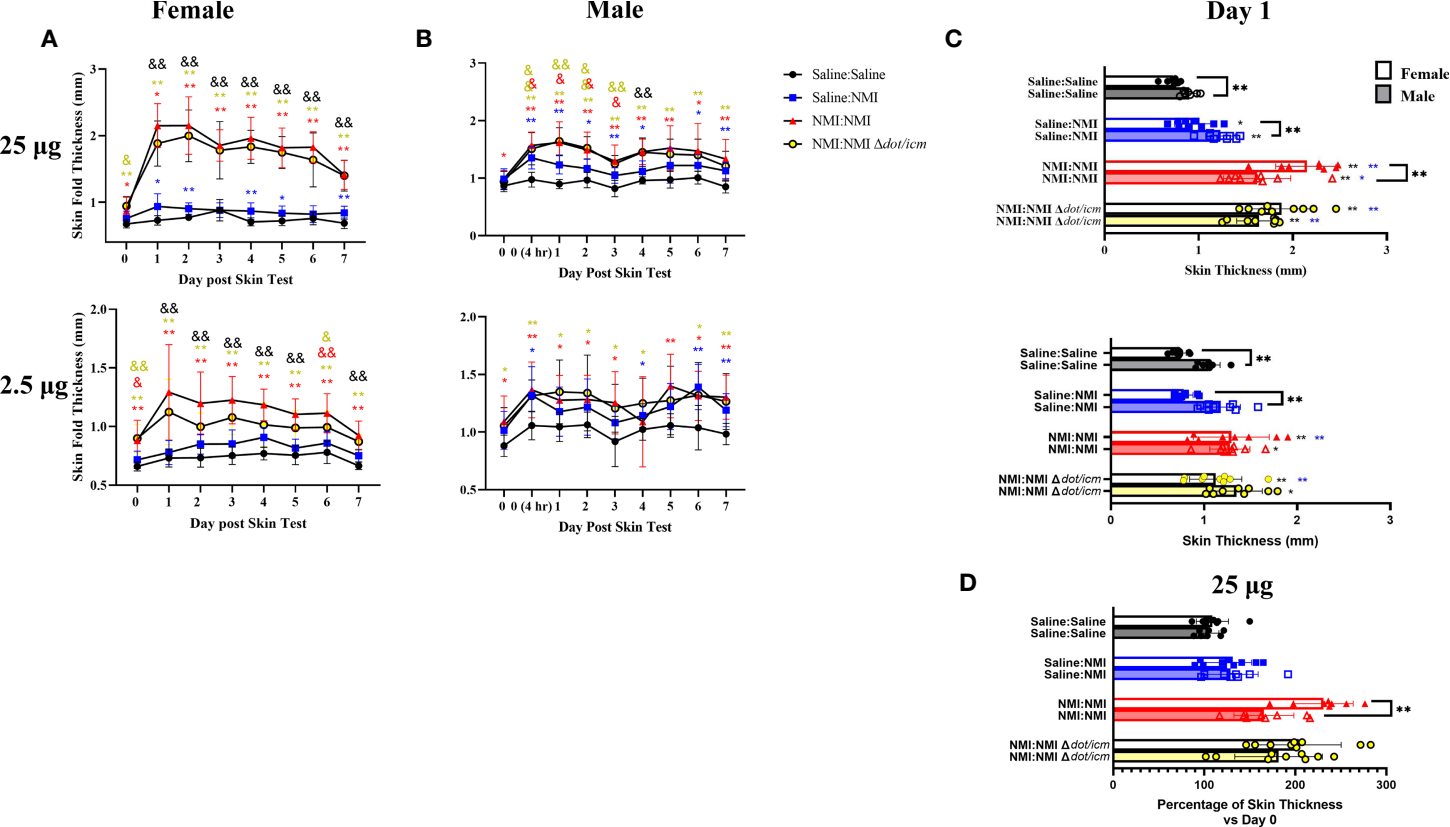
Sensitized female C57Bl/6 mice exhibit robust DTH response. Skin fold thickness as group means is presented for 25 **(A)** and 2.5 **(B)** µg skin testing sites at various times prior to and following skin testing. Individual skin fold thickness values at day one post skin testing are shown in **(C)**. Percent change in day 1 skin fold thickness compared to day 0 skin fold thickness is shown in **(D)**. *p ≤ 0.05, **p ≤ 0.01 compared to Saline : Saline control group means; ^&^ *p* ≤ 0.05, ^&&^ *p* ≤ 0.01 compared to Saline : NMI unsensitized control group means. Symbols correspond to subject group by color.

The repeated sensitization skin testing model was applied to female and male mice to evaluate the influence of sex on this response. An additional sensitized treatment group (NMI : NMI Δ*dot/icm*) was added to evaluate the NMI Δ*dot/icm* WCV as a potentially less reactive vaccine candidate (16). Male mice exhibited generally elevated baseline skin thickness (**Figures 3A, B**), at the peak of the early phase induration response (day 1) male skin thickness was elevated compared to females in Saline : Saline groups (**Figure 3C**). These experiments revealed comparatively increased skin fold thickness at sensitized female skin testing sites, exhibiting statistical significance at day 1 of the 25 µg response in the NMI : NMI group (**Figures 3A–C**). Sensitized male mice experienced increases in skin thickness at 25 µg testing sites with similar kinetics to those of females but these changes were not generally as robust. Regardless, both sensitized male and female mice displayed skin thickness responses that were statistically discernable from that of the negative and unsensitized controls at 25 µg testing sites (**Figure 3A**). Displaying the intensity of the female DTH response, unsensitized and sensitized skin thickness was only discernible in female mice at 2.5 µg testing sites (**Figure 3B**). At the height of induration, sensitized female mice experienced significant increases in skin fold thickness at both skin testing doses compared to negative and unsensitized controls (**Figure 3C**). Male mice experienced significantly increased skin fold thickness at day 1 in both sensitized and unsensitized animals compared to negative controls at 25 µg testing sites. In contrast, 25 µg sensitized NMI : NMI female mice exhibited significantly increased skin fold thickness at day 1 compared to male values (**Figure 3C**). To account for increased male skin thickness at baseline, percent skin thickness change at day 1 compared to initial thickness (day 0) was assessed (**Figure 3D**). 25 µg NMI : NMI female mice retained significantly increased skin thickness compared to their male counterparts. Few significant differences in skin thickness between NMI and NMI Δ*dot/icm* skin tested animals were observed at any point post skin testing.

**Figure 4:**
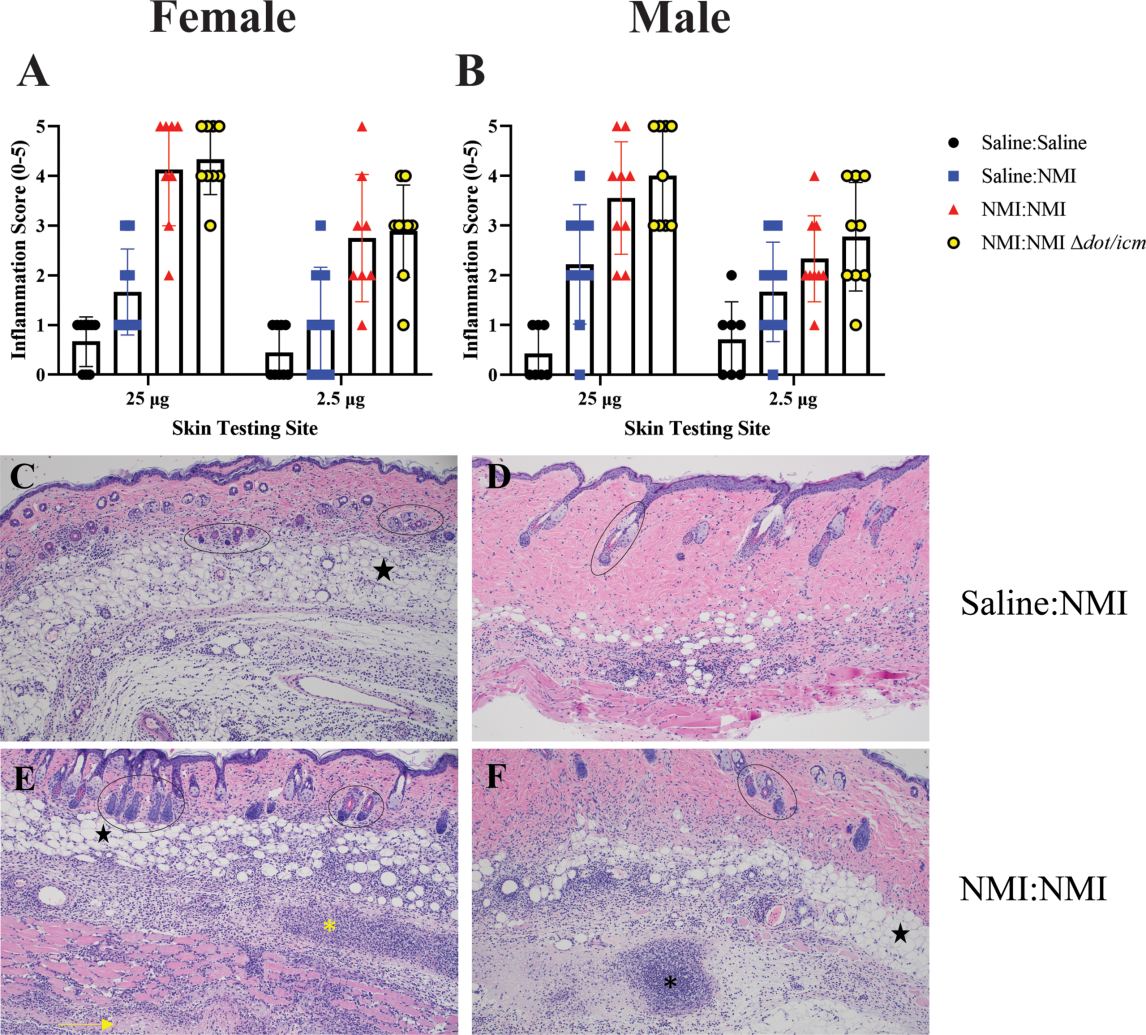
Histologic analysis of female and male skin reveals similar inflammatory responses. Qualitative inflammation scoring was performed on skin biopsies from female **(A)** and male **(B)** mice at 7 days post skin testing. Histologic images are presented for female unsensitized **(C)** and sensitized mice **(E)** and male unsensitized **(D)** and sensitized **(F)** mice. Note the focus of suppurative necrosis in the hypodermis (*) and the necrotic fascia deep to the panniculus m. (->) (bottom left). A focus of suppurative necrosis in the hypodermis of a sensitized male mouse is demarcated (*) in the bottom right. Hair follicles are marked by ovals and black stars indicate adipose tissue. All images are 100x magnification.

Histological analysis at day 7 post skin testing revealed robust increases in skin inflammatory scores in female mice at both skin testing doses (**Figure 4A**). These values appeared to be distinct from that of negative and unsensitized controls, although statistical analysis was not performed due to the subjective nature of the scoring technique. These findings were reflected by that of males (**Figure 4B**). Inflammation extended to the hypodermis, panniculus muscle, and deep fascia in sensitized mice experiencing severe reactions in contrast to that of unsensitized mice (**Figures 4C–F**). Severe inflammation was marked by suppurative necrosis (**Figures 4E, F**). Although robust inflammatory responses were scored in sensitized male mice at both skin testing doses, due to variability in unsensitized animals’ scores, differentiation between unsensitized and sensitized responses was difficult (**Figures 4B, D, F**).

### Local Immune Responses Reveal Increased Immune Cell Numbers in Female Skin

**Figure 5:**
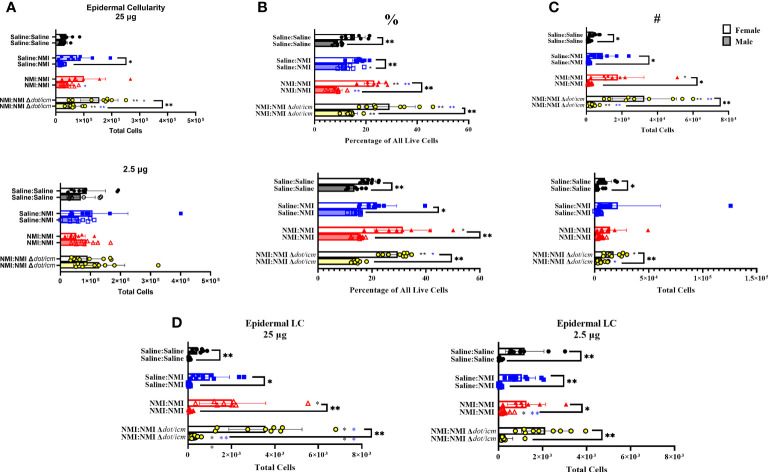
Epidermal cellular immune populations increase in sensitized mice. Cellularity of epidermal single cell suspensions from female and male mice was quantified **(A)** at 7 days post skin testing. Flow cytometric analysis revealed epidermal leukocyte frequency (%) **(B)** and numbers (#) **(C)** along with Langerhans cell (LC) numbers **(D)**. *p ≤ 0.05, **p ≤ 0.01 compared to Saline : Saline (black *) or Saline : NMI (blue *) control group means.

To characterize the local immune response occurring at 7 days post skin testing, epidermal and dermal single cell suspensions were analyzed by flow cytometry. Epidermal cellularity was significantly increased in select male and female sensitized mice at 25 µg sites (**Figure 5A**). Epidermal leukocyte frequency and numbers increased in sensitized female and male animals at 25 µg testing sites (**Figures 5B, C**). Female epidermal leukocyte numbers and frequency were increased in sensitized animals at 2.5 µg sites. This trend was observed in NMI *Δdot/icm* skin tested animals regardless of sex. Female epidermal cellularity was significantly increased over that of males at 25 µg sites in Saline : NMI and NMI : NMI Δ*dot/icm* groups (**Figure 5A**). Leukocyte frequency was significantly increased in all female groups compared to males (**Figure 5B**) and leukocyte numbers were significantly increased in all 25 µg female groups compared to that of males (**Figure 5C**). Langerhans cells (LC) were significantly increased in sensitized female and male mice, with both sensitized female groups exhibiting significantly higher numbers at all sites, regardless of treatment (**Figure 5D**).

**Figure 6:**
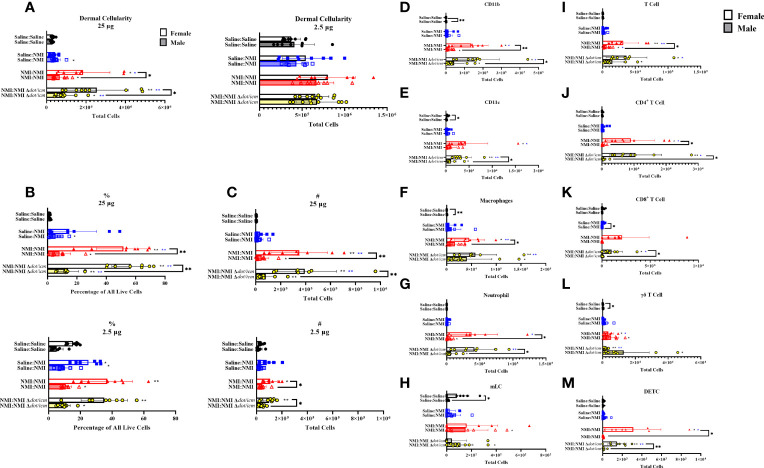
Dermal cellular immune populations increase heterogeneously in female and male sensitized mice. Cellularity of dermal single cell suspensions from female and male mice was quantified **(A)** at 7 days post skin testing. Flow cytometric analysis revealed epidermal leukocyte frequency **(B)** and numbers **(C)**, CD11b^+^ **(D)**, CD11c^+^ **(E)**, macrophage **(F)**, neutrophil **(G)**, migratory Langerhans cell (**H**; mLC), T cell **(I)**, CD4^+^ T cell **(J)**, CD8^+^ T cell **(K)**, γδ T cell **(L)**, and dendritic epithelial T cell (**M**; DETC) numbers. *p ≤ 0.05, **p ≤ 0.01 compared to Saline : Saline (black *) or Saline : NMI (blue *) control group means.

Dermal cellularity was significantly increased in sensitized mice of both sexes at 25 and 2.5 µg testing sites (**Figure 6A**). Like epidermal cellularity, dermal cellularity was significantly increased in sensitized females at 7 days post skin testing compared to males (**Figure 6A**). Dermal lymphocyte frequency and numbers were significantly elevated in sensitized female and male mice at both skin testing sites, with significant elevations in sensitized female values compared to males at 25 µg sites (**Figures 6B, C**). Sporadic increases in leukocyte frequency were observed in the unsensitized control groups in both sexes, albeit to a lower degree (**Figures 6B, C**). Both male and female sensitized mice experienced increases in dermal CD11b^+^ (**Figure 6D**), CD11c^+^ (**Figure 6E**), macrophage (**Figure 6F**), and neutrophil (**Figure 6G**) populations within 25 µg testing sites. Migratory Langerhans cells (mLCs) numbers only appeared to increase in sensitized male dermis samples (**Figure 6H**). At 25 µg sites, sensitized female mice exhibited elevated CD11b^+^, macrophage, and neutrophil numbers compared to their male equivalents. Baseline female values for these populations, along with CD11c^+^ and mLC cells were also significantly increased compared to that of males. No changes due to sex or treatment were observed in dermal myeloid cell populations at 2.5 µg skin testing sites (**Supplementary Figure 4**). Dermal T cell numbers expanded in sensitized male and female mice within 25 µg testing site samples and in male 2.5 µg testing site samples alone (**Figure 6I**). Female dermal T cell expansion within sensitized 25 µg sites corresponded with increases in CD4^+^ T cells(**Figure 6J**), CD8^+^ T cells (**Figure 6K**), γδ T cells (**Figure 6L**), and dendritic epidermal T cells (DETC; **Figure 6M**). In contrast, dermal γδ T cells from male 25 µg skin testing sites were the singular T cell subset to significantly increase in sensitized animals (**Figure 6L**). At 25 µg sites in sensitized animals, T cells, CD4^+^ T cells, CD8^+^ T cells, and DETCs were significantly increased in females compared to males. In contrast, sensitized male mice exhibited significantly increased γδ T cells at baseline (Saline : Saline) compared to females.

### Distal Immune Responses Are Distinct in Female and Male Mice

**Figure 7:**
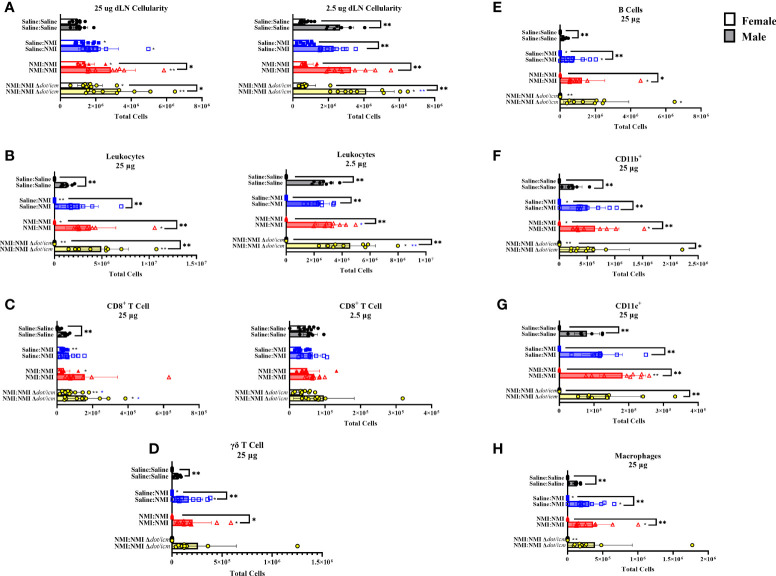
dLN immune populations in female and male mice. Cellularity of dLN single cell suspensions from female and male mice was quantified **(A)** at 7 days post skin testing. Flow cytometric analysis revealed epidermal leukocyte **(B)**, CD8^+^ T cell **(C)**, γδ T cell **(D)**, B cell **(E)**, CD11b^+^ **(F)**, CD11c^+^ **(G)**, and macrophage **(H)** numbers. *p ≤ 0.05, **p ≤ 0.01 compared to Saline : Saline (black *) or Saline : NMI (blue *) control group means.

Both male and female draining lymph node (dLN) cellularity was significantly increased in sensitized mice in brachial lymph nodes draining 25 µg skin testing sites (**Figure 7A**). Unsensitized (25 µg dLN) and NMI Δ*dot/icm* skin tested (2.5 µg dLN) male cellularity was also significantly increased. Despite exhibiting increased skin cellularity during post-skin testing DTH responses, sensitized female mice experienced increased dLN cellularity of a much lower degree compared to their male counterparts (**Figure 7A**). Leukocyte numbers increased in sensitized male and female 25 µg dLN with significant increases only occurring in male inguinal lymph nodes draining 2.5 µg skin testing sites (**Figure 7B**). Male dLNs exhibited significantly elevated leukocyte numbers compared to females among all treatment groups and at baseline. CD8^+^ T cell numbers significantly increased in select treated groups; however, the only sex-specific difference was an increase in CD8^+^ T cells within male 25 µg Saline : Saline groups (**Figure 7C**). Unsensitized and sensitized male mice exhibited higher levels of dLN γδ T cells than females at 25 µg sites (**Figure 7D**). Despite sex-specific differences in numbers, dLN from unsensitized mice of both sexes contained significantly increased numbers of γδ T cells compared to Saline : Saline animals. Non-T cell populations were also profiled in the dLN, revealing no significant alterations in 2.5 µg dLN (**Supplementary Figures 5A–E**). In 25 µg dLN, B cell numbers were increased in unsensitized mice regardless of sex and in select sensitized animals (NMI Δ*dot/icm* – female and all- male; **Figure 7E**). Male mice exhibited significant increases in B cell populations for all 25 µg groups with the exception of NMI : NMI Δ*dot/icm* compared to females. CD11b^+^ dLN cells were increased in sensitized animals, regardless of sex, with male mice expressing significantly increased populations for all 25 µg groups with the exception of NMI : NMI Δ*dot/icm* compared to females (**Figure 7F**). CD11c^+^ dLN numbers were significantly increased for male NMI-skin tested animals alone, with males exhibiting significantly increased CD11c^+^ cells compared to females at all 25 µg sites (**Figure 7G**). Lastly, macrophage numbers were increased in unsensitized and select sensitized dLN, regardless of sex, with males exhibiting significantly increased numbers compared to females at all 25 µg sites with the exception of NMI : NMI Δ*dot/icm* (**Figure 7H**). No changes due to treatment were observed in 2.5 µg dLN among myeloid and γδ T cell populations despite generally higher cell numbers in male samples among all groups compared to females (**Supplementary Figures 5A–E**).

**Figure 8:**
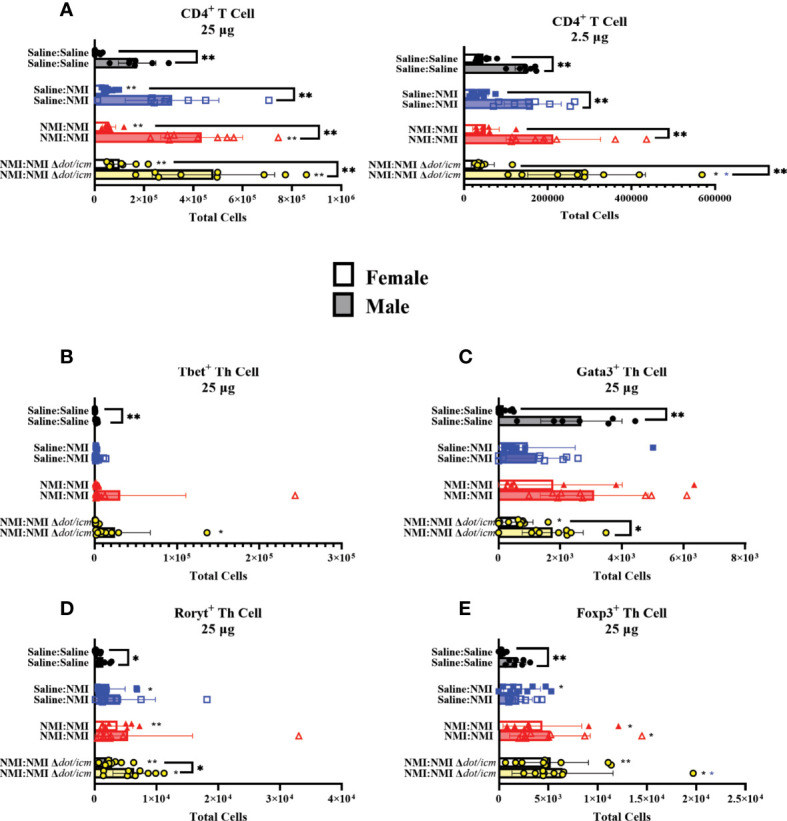
dLN Th populations in female and male mice. Flow cytometric analysis of dLN CD4^+^ T cell **(A)**, Tbet^+^ Th cell **(B)**, Gata3^+^ Th cell **(C)**, Rorγt^+^ Th cell **(D)**, and Foxp3^+^ Th cell **(E)** numbers. *p ≤ 0.05, **p ≤ 0.01 compared to Saline : Saline (black *) or Saline : NMI (blue *) control group means.

dLN CD4^+^ T cell populations were significantly increased in all 25 µg sensitized groups, regardless of sex (**Figure 8A**). Despite this, male mice expressed significantly higher CD4^+^ cell numbers compared to females regardless of treatment or dose. CD4^+^ T cell populations were further analyzed by intracellular transcription factor expression at 7 days following skin testing. Tbet, gata3, rorγt and foxp3 transcription factors are known to influence CD4^+^ helper T cells’ (Th) identity and phenotype (32). Tbet^+^ Th cells appeared to increase following sensitization and during DTH responses (**Figure 8B**). Baseline dLN gata3^+^ Th numbers were elevated at baseline (Saline : Saline) and in sensitized male mice compared to females (**Figure 8C** and **Supplementary Figure 5G**). Both rorγt^+^ and foxp3^+^ Th cell numbers increased in unsensitized (female) and sensitized (both sexes) mice following skin testing in 25 µg dLN (**Figures 8D, E**). No significant alterations in Th transcription factor population numbers were observed in dLN draining 2.5 µg sites (**Supplementary Figures 5F–I**). Baseline Th transcription factor expression (Saline : Saline) was significantly increased in male mice at 25 µg sties compared to females.

**Figure 9:**
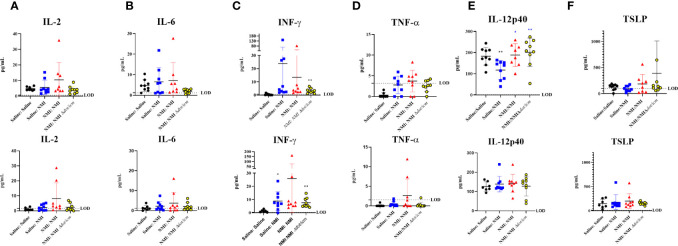
Cytokine analysis reveals sex-specific inflammatory signatures. 25 µg dLN IL-2 **(A)**, IL-6 **(B)**, IFN-γ **(C)**, and TNF-α **(D)** amounts were assessed at 7 days post skin testing. **(E)** IL-12p40 and TSLP **(F)** amounts were assessed at 7 days post skin testing. Values are presented as averages for individual mice as assay was run in duplicate. The assay limit of detection (LOD) is indicated by a dotted line. *p ≤ 0.05, **p ≤ 0.01 compared to Saline : Saline (black *) or Saline : NMI (blue *) control group means.

Multiplex cytokine analysis was performed on cellular lysates from 25 µg dLN at 7 days post skin testing. Although not statistically significant, inflammatory cytokine levels in female unsensitized dLN appeared to generally increase compared to dLN from Saline : Saline mice (**Figures 9A–D**). In unsensitized male mice, interferon-γ (IFN-γ) levels were significantly increased compared to that of Saline : Saline (**Figure 9C**). TNF-α levels in all treated female mice appeared to be higher than that of their male counterparts (**Figure 9D**). Additionally, baseline levels of IL-6 appeared to be higher for female mice than males (**Figure 9B**, Saline : Saline: female: 4.709 pg/mL ± 2.829 SD, male: 1.364 pg/mL ± 1.243 SD). Sensitized mice experienced increased IFN-γ levels in both male and female mice (**Figure 9C**). Many values fell below the assay limit of detection (LOD) for dLN IL-17A (**Supplementary Figure 6A**), IL-17F (**Supplementary Figure 6B**), IL-22 (**Supplementary Figure 6C**), IL-4 (**Supplementary Figure 6D**), IL-9 (**Supplementary Figure 6E**), IL-13 (**Supplementary Figure 6F**), and IL-10 (**Supplementary Figure 6G**) and levels did not appear to significantly increase in any groups compared to Saline : Saline control dLNs. Notably, concentrations of IL-9 (**Supplementary Figure 6E**) and IL-10 (**Supplementary Figure 6G**) were generally higher in male Saline : Saline dLN compared to that of females. In contrast, concentrations of IL-2 (**Figure 9A**) and IL-6 (**Figure 9B**) in female Saline : Saline animals was generally increased compared to that of males.

Select serum cytokine levels were quantified at 7 days *via* multiplex analysis, revealing significantly decreased IL-12p40 levels in unsensitized female mice compared to Saline : Saline animals (**Figure 9E**). In contrast, IL-12p40 levels significantly increased in sensitized animals compared to unsensitized animals. No changes in serum IL-12p40 levels were detected in male mice. Several sensitized female mice expressed high thymic stromal lymphopoietin (TSLP) levels, although group means were not statistically significant compared to Saline : Saline or unsensitized controls (**Figure 9F**). Cytokine data presented in **Figure 9** is also presented in tabular form in **Supplementary Table 2**. C*. burnetii*-specific antibodies were assayed in murine sera at 7 days post skin testing. *C. burnetii*-specific Phase I IgG absorbance was significantly increased in NMI Δ*dot/icm* skin tested male mice (**Supplementary Figure 7A**). *C. burnetii*-specific Phase II IgG absorbance was significantly increased in female sensitized mice and NMI Δ*dot/icm* skin tested male mice (**Supplementary Figure 7B**). Although group means did not display statistical significance, unsensitized and male NMI skin tested animals also appeared to have generally increased *C. burnetii*-specific Phase II IgG reactivity. *C. burnetii*-specific Phase II IgM absorbance was significantly increased in unsensitized mice following initial skin testing regardless of sex (**Supplementary Figure 7C**).

## Discussion

Post vaccination DTH modeling is a crucial tool in the development of safe human vaccines. Not only can these models be used to screen vaccine candidates, they can be used to gain insight into the immunologic mechanisms driving ensuing allergic responses. In humans, the Q fever WCV DTH reaction is generally biphasic, with an early response that resembles Tuberculin-type DTH and a late phase characterized by the formation of granulomas/abscesses (13). Tuberculin reactions are generally elicited following intradermal injection of a *Mycobacterium tuberculosis* antigen preparation and are marked by dermal cellular infiltration and peak induration from 24-72 hours, depending on the host species (33). For example, guinea pigs exhibit a neutrophilic infiltrate in contrast to the primarily monocytic nature of that of humans (1). Granulomatous reactions are sometimes described as a distinct subtype of DTH and it appears that development is contingent upon enhanced antigen persistence (e.g., insolubility), dose, and ensuing macrophage involvement (34). Granulomatous infiltrates are typically characterized by epithelioid macrophages or fused macrophages (e.g., multinucleated giant cells) along with lymphocytes (34). Due to its reversable and transient nature, early-phase DTH has been described as “non-destructive”, while late-phase granulomatous reactions have been described as “destructive” (35). For *M. tuberculosis* antigen-induced DTH responses, both early and late manifestations were elicitation dose-dependent (35). The degree of shared immunologic mechanisms driving both responses is unclear. Although frequently presented as a distinct reaction, granulomatous occurrences may be related to/preceded by other DTH reactions, such as tuberculin-type reactions, given the similarities of each cellular infiltrate, cytokine milieu, and shared antigen. It is reasonable to speculate that the inflammatory environment established during early-phase responses could promote granuloma formation. Indeed, the early-phase tuberculin DTH response is considered an important event in the sequence of *M. tuberculosis* granuloma formation (36). These concurrent responses are also exhibited in a murine hapten-polyacrylamide bead DTH model, with maximal induration occurring in mouse footpads 24 hours post challenge followed by early granuloma formulation at 72-96 hours (37, 38).

Late-phase granulomatous responses have been the focus of previous post Q fever vaccination DTH models; however, human Q fever WCV studies indicate the consistent occurrence of an early-phase DTH response in vaccinated and skin tested individuals. To address this disparity, we developed a murine early-phase post Q fever vaccination DTH model in C57Bl/6J mice. Repeated sensitization events and a 14-day challenge interval were necessary for a sufficiently robust early-phase DTH response following sensitization and skin testing. This model produced robust DTH responses in WCV sensitized mice characterized by increased skin thickness peaking at 24 hours post skin testing. At 7 days post skin testing, sensitized skin sites were characterized by inflammation consisting of macrophages, leukocytes, and occasional neutrophils. Despite mild inflammatory responses in unsensitized animals, sensitized animals exhibited significantly elevated DTH responses compared to unsensitized controls. These responses were most distinct in C57Bl/6J mice compared to BALB/cJ and SKH-1 strains. Given that C57Bl/6J is a Th1-biased strain (30) and this response is likely Th1-mediated (23), these results are not surprising. Although SKH-1 mice exhibited significantly increased DTH responses (as measured by skin fold thickness) in sensitized animals at 25 µg sites, Saline : Saline mice also displayed notable pyogranulomatous inflammation. This baseline inflammation is a potential confounding factor in the interpretation of results; therefore, we do not advise the use of this strain in our model. This was not reported by Fratzke, et al. who suggested the SKH-1 strain was suitable for a single-sensitization model (23).

While initial studies were conducted in female mice, we sought to examine the effect of sex on this response. Generally, female skin thickness increased to a greater degree following skin testing than that of males and this finding was statistically significant at the peak of DTH (day 1 post skin testing) in the 25 µg NMI : NMI group despite initial increased male skin thickness. Additionally, epidermal and dermal cellularity were higher in sensitized females at 7 days post skin testing compared to males. In contrast, histopathologic inflammation scores were similar for both sexes. Inflammation scores were based on skin biopsies collected at 7 days post skin testing, after the early-phase response has “peaked” in severity. Therefore, these metrics may better reflect developing granulomatous responses, likely due to antigen persistence at the injection site (36), which may not be affected by the animal’s sex.

At 25 µg skin testing sites, epidermal cellularity and leukocyte populations expanded in sensitized female and male mice, with female values exhibiting statistical significance above that of males. Increasing epidermal leukocytes appeared to be comprised of LC, likely acting as antigen presenting cells (APC) upon migration to the dermis and dLN. Dermal leukocyte frequencies increased for all sensitized groups, regardless of sex; however, skin tested female mice exhibited higher frequencies and cell numbers compared to that of males at 25 µg sites. Dermal immune cell profiling at day 7 post skin testing revealed increases in several myeloid cell populations in sensitized mice, representing a variety of putative APCs (macrophages, migratory LC, and CD11c^+^ cells) and granulocytes (neutrophils), reflecting histological findings at this time point. 25 µg sensitized female mice displayed significantly higher CD11b^+^, macrophage, and neutrophil populations compared to males. Dermal T cell populations were increased in sensitized mice at 25 µg sites regardless of sex and at 2.5 µg sites in male mice. Again, female T cell numbers in sensitized mice were of a much higher magnitude than that of males. The composition of male and female dermal T cell populations at 25 µg sites were distinct. Female CD4^+^, CD8^+^, γδ, and DETC populations increased in sensitized mice while only γδ T cells increased in male dermal samples. These cells are likely dermal-resident γδ T cells (39) as DETC numbers remained steady. Male mice expressed significantly higher numbers of dermal γδ T cells at baseline (Saline : Saline) than females. These uniquely expressed cell populations are likely involved in mediating immune responses that may be sex specific. For example, in male mice dermal γδ T cells may be serving a regulatory role (40) and the lack of potentially pathogenic CD4^+^ and CD8^+^ subsets align with male skin thickness outcomes.

In the dLN, many immune cell populations were increased in unsensitized animals, indicating that this was a principal site of immune activity during early sensitization (7 days post skin testing). In contrast to skin cellularity, male dLN cellularity was higher than females’ in skin tested animals, regardless of dose. Overall, dLN T cell composition was similar among mice, regardless of sex, with increases in CD4^+^ and CD8^+^ T cells. Only male γδ T cells were increased in unsensitized and sensitized animals in 25 µg draining lymph nodes and this population was significantly increased in male unsensitized and sensitized (NMI : NMI) mice. These cells are likely migrating dermal-resident γδ T cells, as this putative population also expanded in male dermal skin testing sites. APC numbers, B cell numbers, CD4^+^ T helper (Th) cell transcription factor expression analysis, and cytokine analysis indicated an immune activated and inflammatory environment in 25 µg dLN in skin tested mice. Increases in dLN IFN-γ in unsensitized, skin tested mice supports prior findings that a Th1-type response is important in driving WCV-induced DTH (23).

There is precedence for divergent DTH and immune responses observed between female and male mice. Generally, female mice display augmented allergic responses (41–44) and adult female humans are thought to mount stronger innate and adaptive immune responses than males, leading to increased susceptibility to autoimmune and allergic diseases (45). Further, post-vaccination injection site reactions, some of which are not DTH, are reported more frequently among females (46) (47, 48).Indeed, female mice displayed augmented DTH responses as represented by increased skin thickness in response to *C. burnetii* WCV sensitization and elicitation. Skin and dLN immune environments were also unique in each sex both at baseline and following skin testing. We speculate that male mice exhibit unique innate regulatory mechanisms which result in dampened DTH responses. Antigen persistence is likely a major driving factor of granuloma formation, promoting delayed (>7 days post skin testing) responses, and this facet of the post vaccination response may not be impacted as dramatically by sex-related factors as that of early-phase DTH, given our findings. Regardless, potential mechanisms responsible for diminished male early-phase DTH responses include intrinsic immune features present in negative control (Saline : Saline) mice. We detected elevated dLN gata3^+^ Th cell numbers in Saline : Saline male dLNs along with generally higher concentrations of IL-10. Gata3^+^ Th2 cells likely have a regulatory effect during Th1 biased DTH responses *via* IL-4 secretion (49, 50). While local cytokine responses were not measured in this study, generally increased numbers of dLN gata3^+^ Th cells at baseline and post skin testing may indicate a role for this regulatory pathway in male mice. Additionally, elevated dLN IL-10 concentrations were observed in male mice at baseline and during sensitization (e.g., in unsensitized mice following skin testing) compared to that of females. This cytokine is known to suppress DTH during both the sensitization and elicitation phases (51) and expression in the dLN during sensitization would likely have an important regulatory effect on T cell differentiation, potentially suppressing inflammatory Th cytokine secretion and reshaping the dLN milieu leading to dampened DTH responses following elicitation (52). Dendritic cells (DC), macrophages, and T_regs_ are major sources of IL-10 (52); in our model, these cell types exhibit higher numbers in male dLN compared to females at baseline and during sensitization, serving as potential sources of this cytokine. In addition to IL-10, male dLN IL-9 levels were generally higher than that of females at baseline and during sensitization. In the skin, IL-9 is thought to have a pathogenic role in DTH (53) but the role of this cytokine in the dLN during DTH has not been elucidated. It is not likely that expression of IL-9 contributes to negative regulation of the DTH response in our model, rather, it may represent an increased population of Th9 cells that may home to the skin and contribute to inflammation (53). Female mice appeared to express higher dLN levels of the inflammatory cytokines IL-6 and TNF-α during sensitization compared to males. IL-6 is an important factor in determining the magnitude of ensuing DTH responses when expressed in the dLN during sensitization (42). These two cytokines also appear to be expressed at higher baseline levels in female dLN compared to males in our model. Thus, Female DTH responses may be influenced by increased IL-6 and TNF-α expression.

Local immune responses in male and female mice were similar in composition but several important observations may relate to the distinct magnitude of early-phase DTH intensity observed among the two sexes. Male mice exhibited lower leukocyte frequency during sensitization and elicitation at both skin testing sites compared to females. Within the leukocyte population, all T cell populations enumerated were significantly increased in sensitized female mice, while this was only true for γδ T cells in males. Although this data was collected 7 days following skin testing and may somewhat represent the developing granulomatous response, this finding may indicate a regulatory mechanism for γδ T cells or may simply reflect the absence of potentially pathogenic subsets through an alternative mechanism, but this remains to be investigated. Female mice also express higher numbers of epidermal LC during sensitization and elicitation compared to males. LCs can serve as critical APCs during DTH (1) and female mice may exhibit more robust antigen presentation and T cell-mediated DTH responses as a result. Although the specific mechanism(s) of sex dependent DTH magnitude are unknown, the role of sex in this model is likely important when considering the human condition. Sex differences in responses to *C. burnetii* infection have been documented, with 17-β-estradiol likely playing a protective role. Additionally, sex hormones have been shown to influence macrophage responses to *C. burnetii in vitro* (48). These findings suggest that host sex hormones may be involved in the sexually dimorphic DTH responses demonstrated here. Although infection and DTH immune responses appear to share some conserved immunologic mechanisms, these states are not necessarily analogous. Thus, the influence of sex hormones on our findings remains to be proven. In humans, the influence of sex on post Q fever vaccination DTH responses is unknown, but this data suggests that it should be an important consideration for vaccine development and evaluation.

This work presents a murine post-Q fever vaccination early-phase DTH model which reveals sexually dimorphic immune responses at 7 days post skin testing. This model will likely be useful for the routine evaluation of Q fever vaccine candidates in biosafety level 2 settings. The NMI Δ*dot/icm* WCV vaccine candidate did not cause a response that was significantly different than wild type NMI WCV in this model, despite exhibiting potentially reduced reactogenicity in the guinea pig model (16). It is important to note that the guinea pig model primarily reflects a granulomatous response and does not capture the early-phase response described here. By recapitulating the early-phase DTH response, this model may better represent the human condition in which severe granulomatous responses are preceded by early-phase DTH responses (13). In addition, it is likely that granuloma or abscess formation would occur in this model, if extended, given the histological makeup of skin testing sites at 7 days post skin testing (e.g., suppurative necrosis). DTH response magnitude was impacted by mouse sex, raising important considerations for Q fever and bacterial WCV vaccine development. Local and distal immune factors, such as γδ T cells and select cytokines, are potentially involved in the mediation of altered DTH responsiveness between sexes. This work provides insight into *C. burnetii* WCV-related early-phase DTH responses and reveals the influence of sex as a factor in the severity and composition of the response (**Supplementary Figure 8**), introducing a useful early-phase DTH model that will contribute to our understanding of this response and to the development of an improved Q fever vaccine.

## Data Availability Statement

The original contributions presented in the study are included in the article/**Supplementary Material**. Further inquiries can be directed to the corresponding author.

## Ethics Statement

The animal study was reviewed and approved by Rocky Mountain Laboratories Institutional Animal Care and Use Committee.

## Author Contributions

PB, MT, and CML conceptualized the experiments and wrote the manuscript. PB, MT, DC, CS, CR, and CML performed experiments and analyzed data. All authors contributed to the manuscript, assisted with editing, and approved the submitted version.

## Funding

This work was supported by the Intramural Research Program of the National Institutes of Health, National Institute of Allergy and Infectious Disease (ZIAAI001331).

## Conflict of Interest

The authors declare that the research was conducted in the absence of any commercial or financial relationships that could be construed as a potential conflict of interest.

## Publisher’s Note

All claims expressed in this article are solely those of the authors and do not necessarily represent those of their affiliated organizations, or those of the publisher, the editors and the reviewers. Any product that may be evaluated in this article, or claim that may be made by its manufacturer, is not guaranteed or endorsed by the publisher.
