# Supplementary material for: Murine Q Fever Vaccination Model Reveals Sex Dimorphism in Early Phase Delayed-Type Hypersensitivity Responses
Source: Front Immunol. 2022 Jun 15;13:894536. doi: 10.3389/fimmu.2022.894536 (PMC9241443; doi:10.3389/fimmu.2022.894536)
Supplement: Supplementary file 1 [file DataSheet_1.docx]

Supplementary Table 1 – Flow Cytometric Panels

| Marker | Fluorophore | Clone | Manufacturer | Cat# | Staining Panel |
| --- | --- | --- | --- | --- | --- |
| B220 | BUV615 | RA3-6B2 | BD | 751580 | 1 |
| CD3 | BUV661 | 145-2C11 | BD | 750638 | E, D |
| CD4 | BUV395 | GK1.5 | BD | 563790 | D, 1, 2 |
| CD4 | BV785 | RM4-5 | BioLegend | 100552 | 1, 2 |
| CD8 | BV510 | 53-6.7 | BioLegend | 100752 | D, 1, 2 |
| CD11b | BB515 | M1/70 | BD | 564454 | E, D, 1 |
| CD11c | BUV563 | HL3 | BD | 749091 | E, D, 1 |
| CD19 | AF700 | 6D5 | BioLegend | 115528 | 1 |
| CD44 | BUV496 | IM7 | BD | 741057 | 2 |
| CD45 | PerCP Cy5.5 | 30-F11 | BioLegend | 103132 | E, D, 1 |
| CD62L | APC Cy7 | MEL-14 | BioLegend | 104428 | 2 |
| CD62L | BUV737 | MEL-14 | BD | 612833 | 2 |
| CD69 | AF700 | H1.2F3 | BioLegend | 104539 | E, D |
| CD103 | PE | 2E7 | BioLegend | 121406 | E, D |
| CD207/Langerin | APC | 4C7 | BioLegend | 144206 | E, D |
| F4/80 | BV650 | BM8 | BioLegend | 123149 | D, 1 |
| Foxp3 | BV421 | MF-14 | BioLegend | 126419 | D, 2 |
| Gata3 | AF488 | 16E10A23 | BioLegend | 653808 | 2 |
| I-A/I-E | PE Cy5 | M5/114.15.2 | BioLegend | 107612 | E, D |
| Live/Dead | Zombie Yellow | - | BioLegend | 423104 | All |
| Ly6G | PE Cy7 | 1A8 | BioLegend | 127618 | D |
| Rorγt | AF647 | Q31-378 | BD | 562682 | 2 |
| Tbet | PE Dazzle™ 594 | 4B10 | BioLegend | 644828 | 2 |
| TCRγδ | BV605 | GL3 | BioLegend | 118129 | E, D, 1 |
| XCR1 | BV785 | ZET | BioLegend | 148225 | D |

Epidermis (E), dermis (D), dLN 1 (1), dLN 2 (2)

Supplementary Table 2 – Cytokine Data Summary

dLN

|  | Saline:Saline | | Saline:NMI | | NMI:NMI | | NMI:NMI ∆*dot/icm* | |
| --- | --- | --- | --- | --- | --- | --- | --- | --- |
|  | Female | Male | Female | Male | Female | Male | Female | Male |
| IL-2 | 4.701 ± 0.375 | 1.253 ± 0.4 | 5.588 ± 1.544 | 2.272 ± 0.668 | 10.46 ± 3.947 | 8.218 ± 3.671 | 3.937 ± 0.787 | 2.45 ± 0.911 |
| IL-6 | 4.809 ± 0.943 | 1.364 ± 0.47 | 6.503 ± 2.298 | 2.263 ± 0.767 | 7.143 ± 3.109 | 3.766 ± 1.771 | 1.973 ± 0.329 | 2.109 ± 0.626 |
| INF-γ | 0.393 ± 0.195 | 1.303 ± 0.404 | 23.84 ± 15 | 8.845 ± 2.386 | 13.51 ± 9.792 | 25.9 ± 17.11 | 3.218 ± 0.607 | 7.579 ± 1.209 |
| TNF-α | 0.34 ± 0.238 | 0.126 ± 0.126 | 2.787 ± 0.708 | 0.483 ± 0.209 | 3.715 ± 0.873 | 2.617 ± 1.406 | 2.572 ± 0.45 | 0.291 ± 0.226 |

Serum

|  | Saline:Saline | | Saline:NMI | | NMI:NMI | | NMI:NMI ∆*dot/icm* | |
| --- | --- | --- | --- | --- | --- | --- | --- | --- |
|  | Female | Male | Female | Male | Female | Male | Female | Male |
| IL-12p40 | 182.5 ± 14.04 | 126.3 ± 9.222 | 117.3 ± 14.98 | 138.8 ± 13.45 | 188.2 ± 19.06 | 142.5 ± 15.55 | 201.2 ± 22.5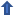 | 127.9 ± 16.92 |
| TSLP | 125.3 ± 19.58 | 146.9 ± 35.54 | 93.77 ± 17.49 | 177.5 ± 51.79 | 186.3 ± 63.53 | 200.3 ± 50.71 | 389.2 ± 207.8 | 153 ± 10.88 |

Table 2: Cytokine values are presented as group mean value (pg/mL) ± SEM. Statistical significance (p<0.05) is indicated by arrows: significant compared to saline:saline; significant compared to saline:NMI.

Supplementary Figure Legends

Figure 1 - Single sensitization model is not sufficient to induce a measurable early-phase DTH response in C57Bl/6 mice

Murine single (A) sensitization model is outlined. Syringes indicate saline or WCV injections and tubes indicate blood collection. Skin thickness kinetics are displayed in B.

Figure 2- Flow cytometric gating strategy

Epidermal (A), dermal (B), dLN 1 (C), and dLN 2 (D) flow cytometric gating strategies are presented. In all panels, leukocytes are defined as singlets, all cells, live, CD45^+^. From this population the following subpopulations were defined: T cells (CD3^+^), CD4^+^ Th cells (CD3^+^, CD4^+^), CD8^+^ Th cells (CD3^+^, CD8^+^), γδ T cells (CD3^+^, TCR γδ^+^), DETC (CD3^+^, TCR γδ^+^, CD103^+^), LC (CD11b^+^, CD207^+^, I-Ab^+^), neutrophils (CD11b^+^, Ly6G^+^), macrophages (CD11b^+^, F4/80^+^), and B cells (CD19^+^, B220^+^). For D, negative control populations (fluorescence minus one control) were shown above positively stained populations.

Figure 3- Inflammation scoring scheme

Qualitative inflammation scoring was performed on skin biopsies from mice at 7 days post skin testing. The resultant inflammation scoring scheme along with representative images are described. All images are at 100X magnification.

Figure 4 – Dermal flow cytometric analysis of myeloid cell populations at 2.5 µg skin testing sites

Flow cytometric analysis of CD11b^+^ (A), CD11c^+^ (B), macrophage (C), migratory Langerhans cell (D; mLC), and neutrophil (E) populations at 7 days post skin testing.

Figure 5 – 2.5 µg dLN immune populations in female and male mice

Flow cytometric analysis of Tbet^+^ Th cell (A), Gata3^+^ Th cell (B), Rorγt^+^ Th cell (C), Foxp3^+^ Th cell (D), γδ T cell (E), B cell (F), CD11b^+^ (G), CD11c^+^ (H), and macrophage (I) numbers.

Figure 6 – Cytokine analysis reveals sex-specific inflammatory signatures

25 µg dLN IL-17A (A), IL-17F (B), IL-22 (C), IL-4 (D), IL-9 (E), IL-13 (F), and IL-10 (G) values were assessed at 7 days post skin testing. Values are presented as averages for individual mice as assay was run in duplicate. The assay limit of detection (LOD) is indicated by a dotted line.

Figure 7 – Serum *Coxiella burnetii*-specific antibody assessment

Phase I (A) and Phase II (B) *C. burnetii*-specific IgG and Phase II IgM (C) expression was quantified in mouse sera 7 days post skin testing. Values are presented as averages for individual mice as assay was run in duplicate. * *p* ≤ 0.05, ** *p* ≤ 0.01 compared to Saline:Saline control group means.

Figure 8 – Graphical Abstract

Major findings of the study are summarized here. A murine early-phase DTH model was developed via repeated Q fever vaccinations (sensitization) followed by intradermal vaccination (elicitation). Compared to males, female C57Bl/6 mice exhibited increased DTH responses and skin effector cell numbers, including Langerhans cells, neutrophils, macrophages, CD4^+^ T cells and CD8^+^ T cells. In contrast, male mice experienced lower magnitude DTH responses, marked by decreased effector cell numbers in the skin and increased dermal/draining lymph node γδ T cell numbers.
