# Supplementary figures and images for: Murine Q Fever Vaccination Model Reveals Sex Dimorphism in Early Phase Delayed-Type Hypersensitivity Responses
Source: Front Immunol. 2022 Jun 15;13:894536. doi: 10.3389/fimmu.2022.894536 (PMC9241443; doi:10.3389/fimmu.2022.894536)

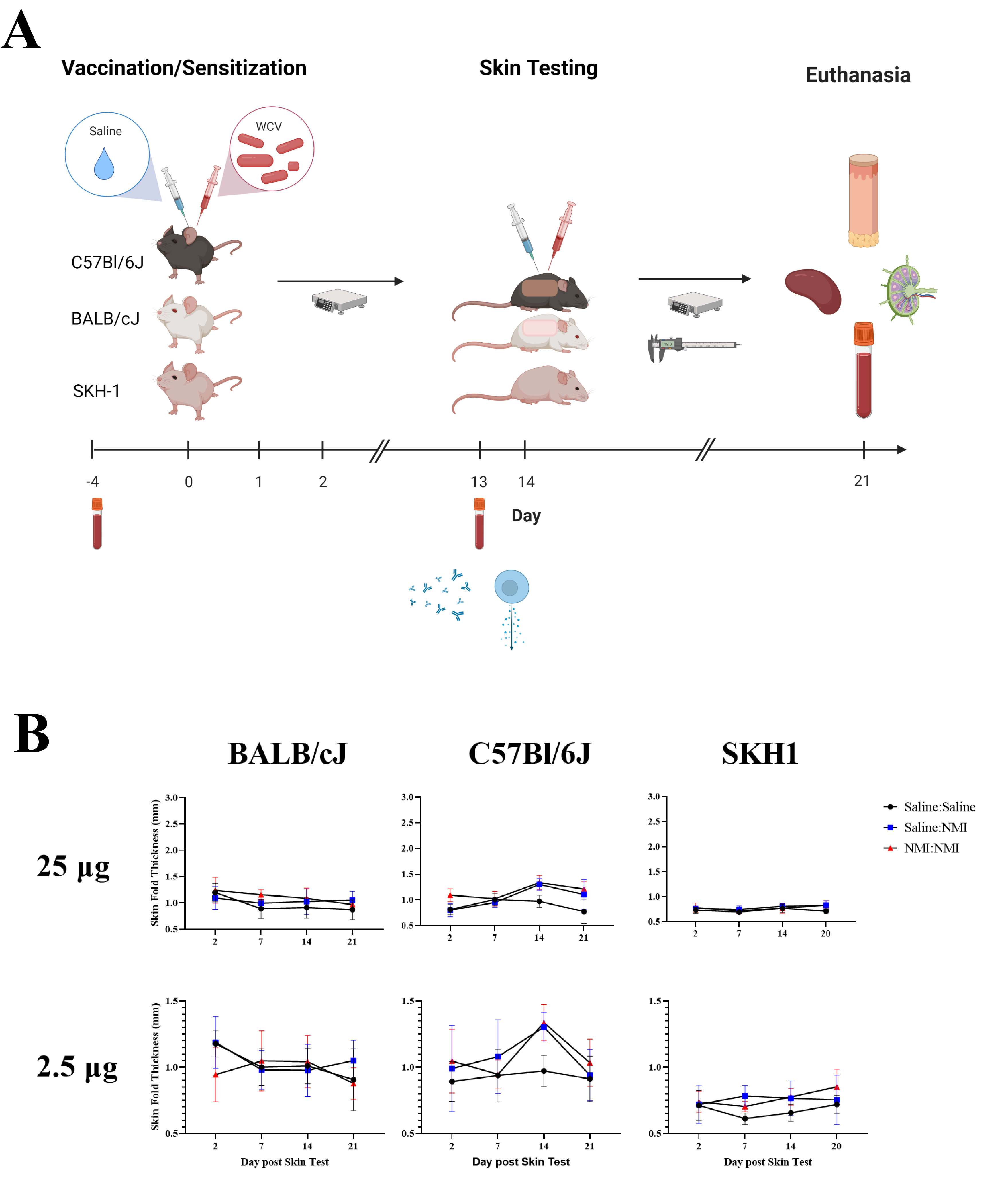

Supplement: Supplementary file 2 [file Image_1.tif]

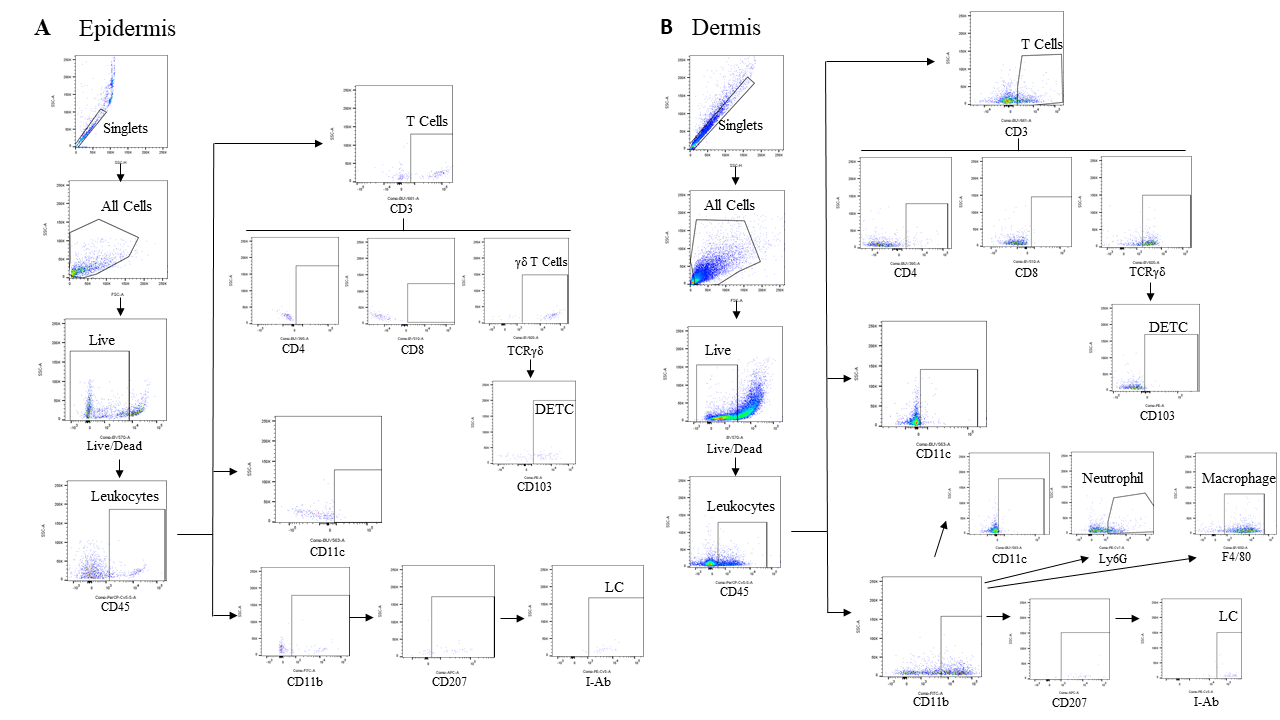

Supplement: Supplementary file 3 [file Image_2.png]

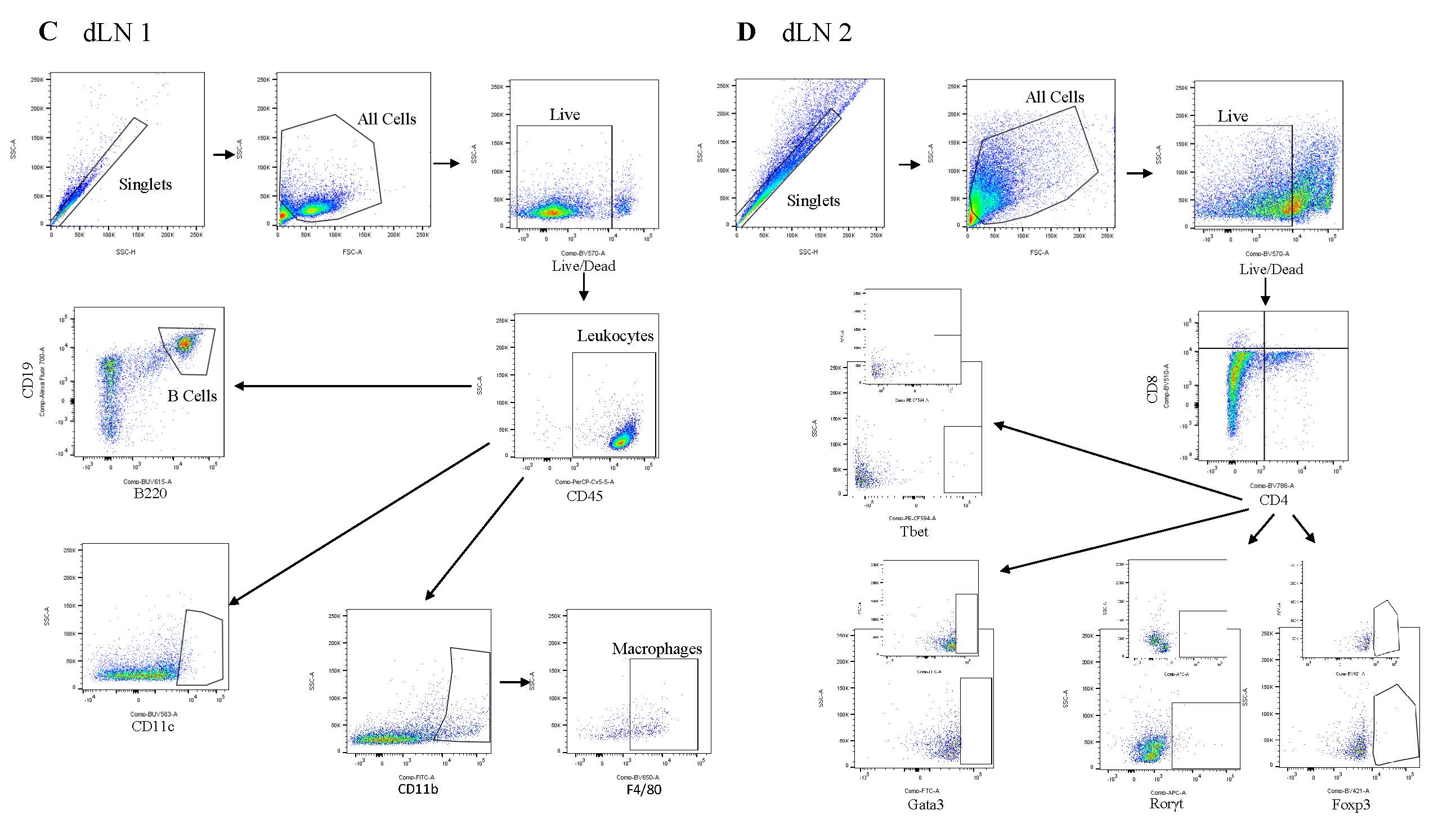

Supplement: Supplementary file 4 [file Image_3.jpeg]

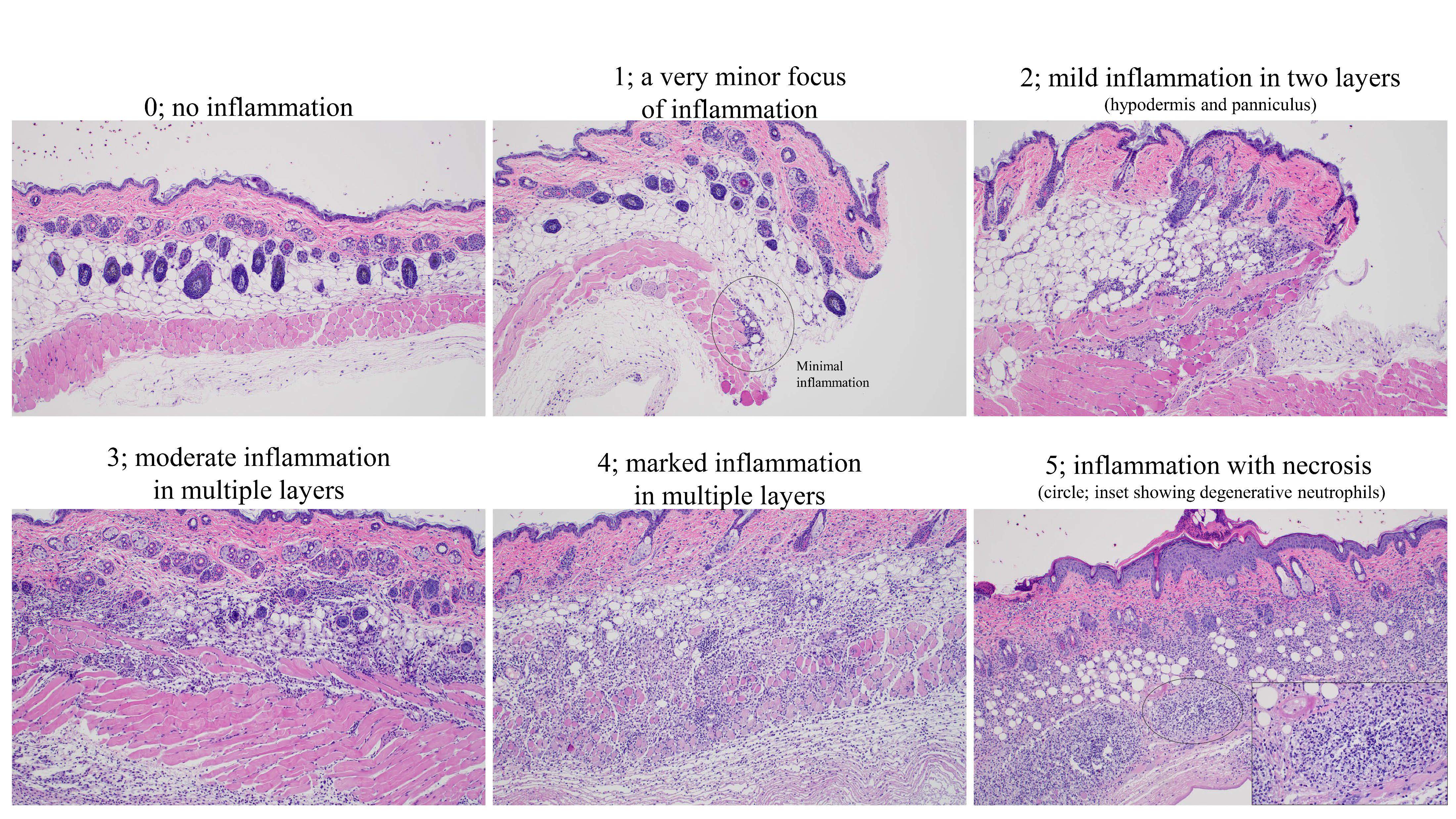

Supplement: Supplementary file 5 [file Image_4.jpeg]

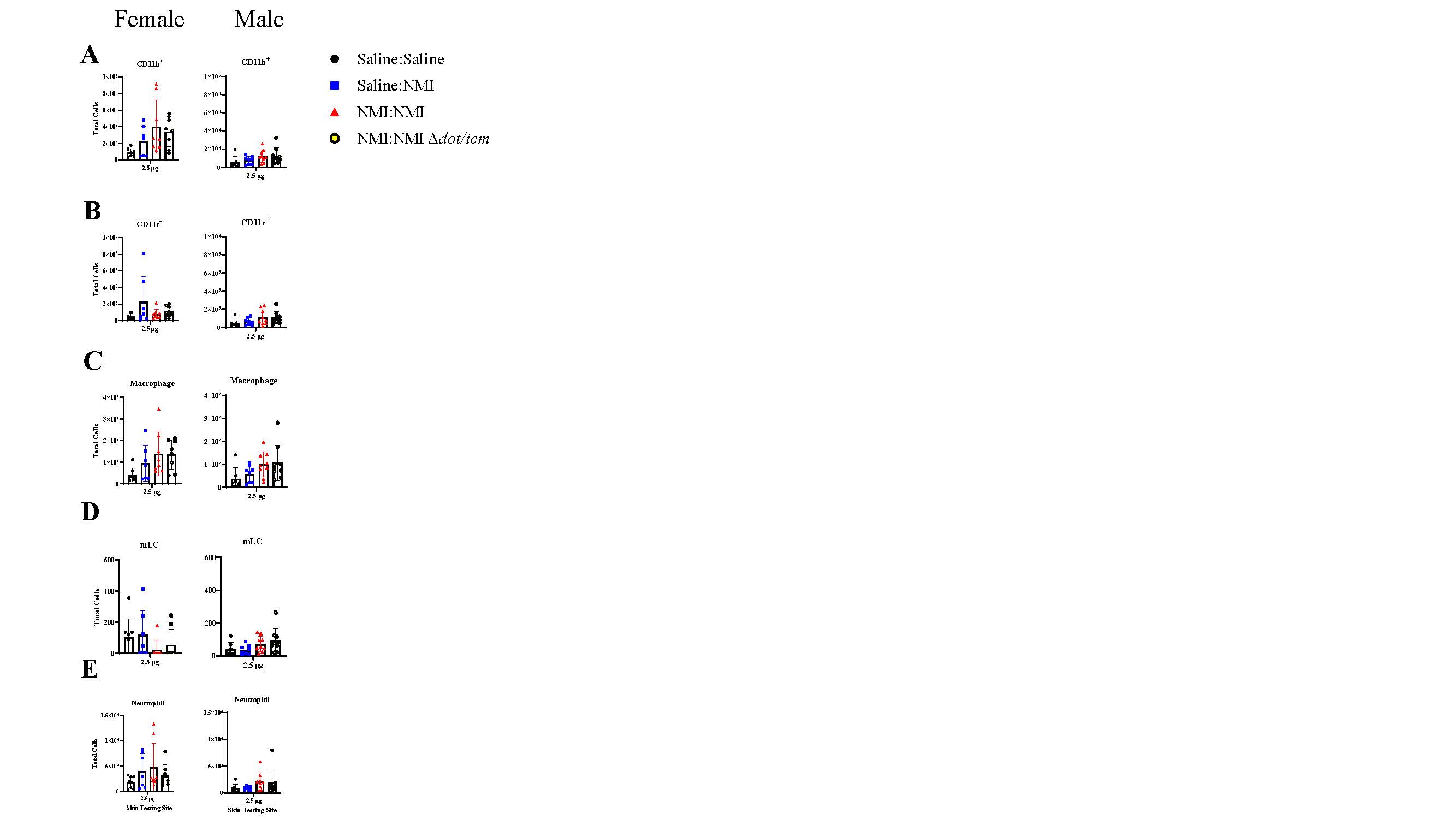

Supplement: Supplementary file 6 [file Image_5.jpeg]

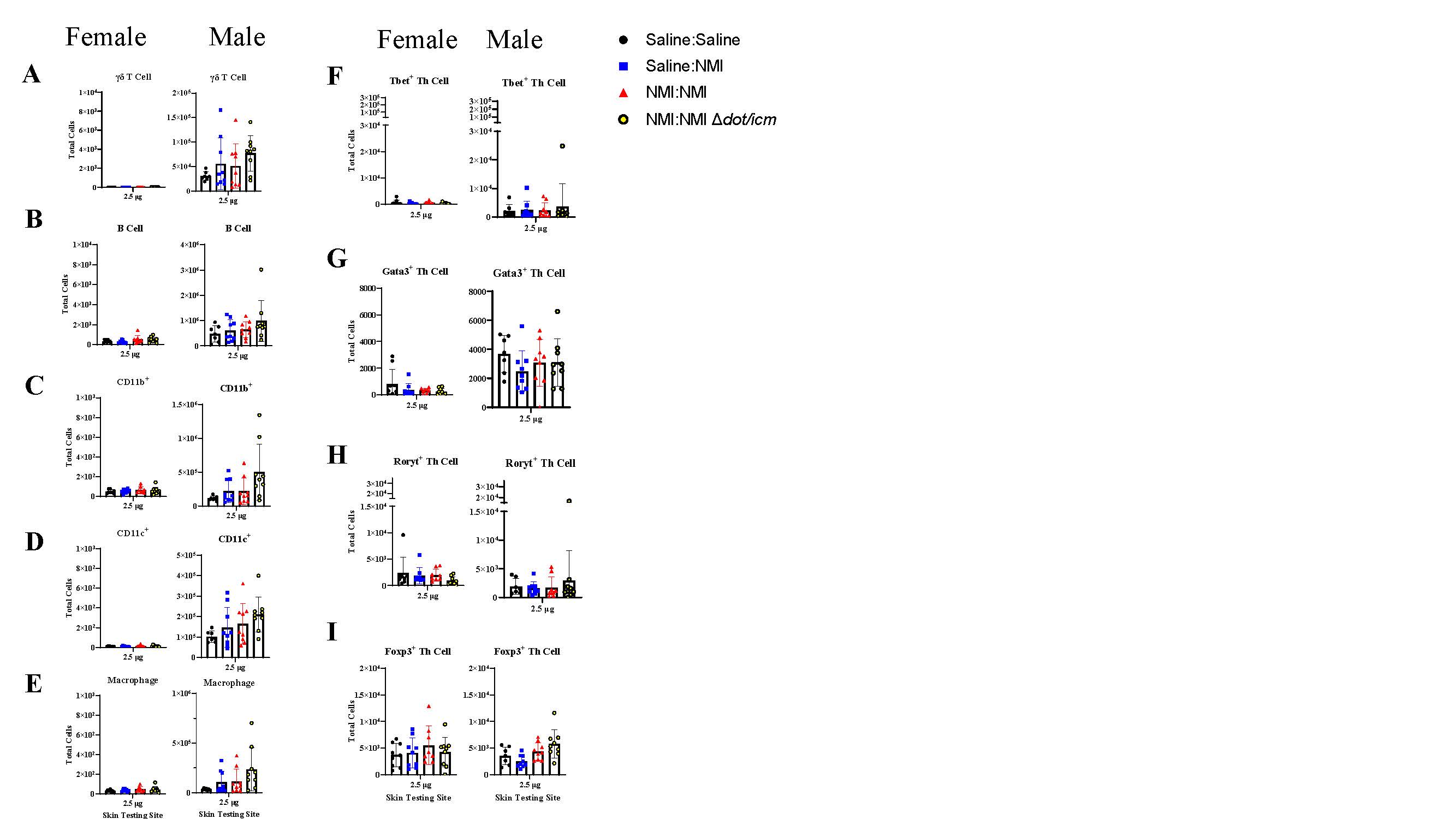

Supplement: Supplementary file 7 [file Image_6.jpeg]

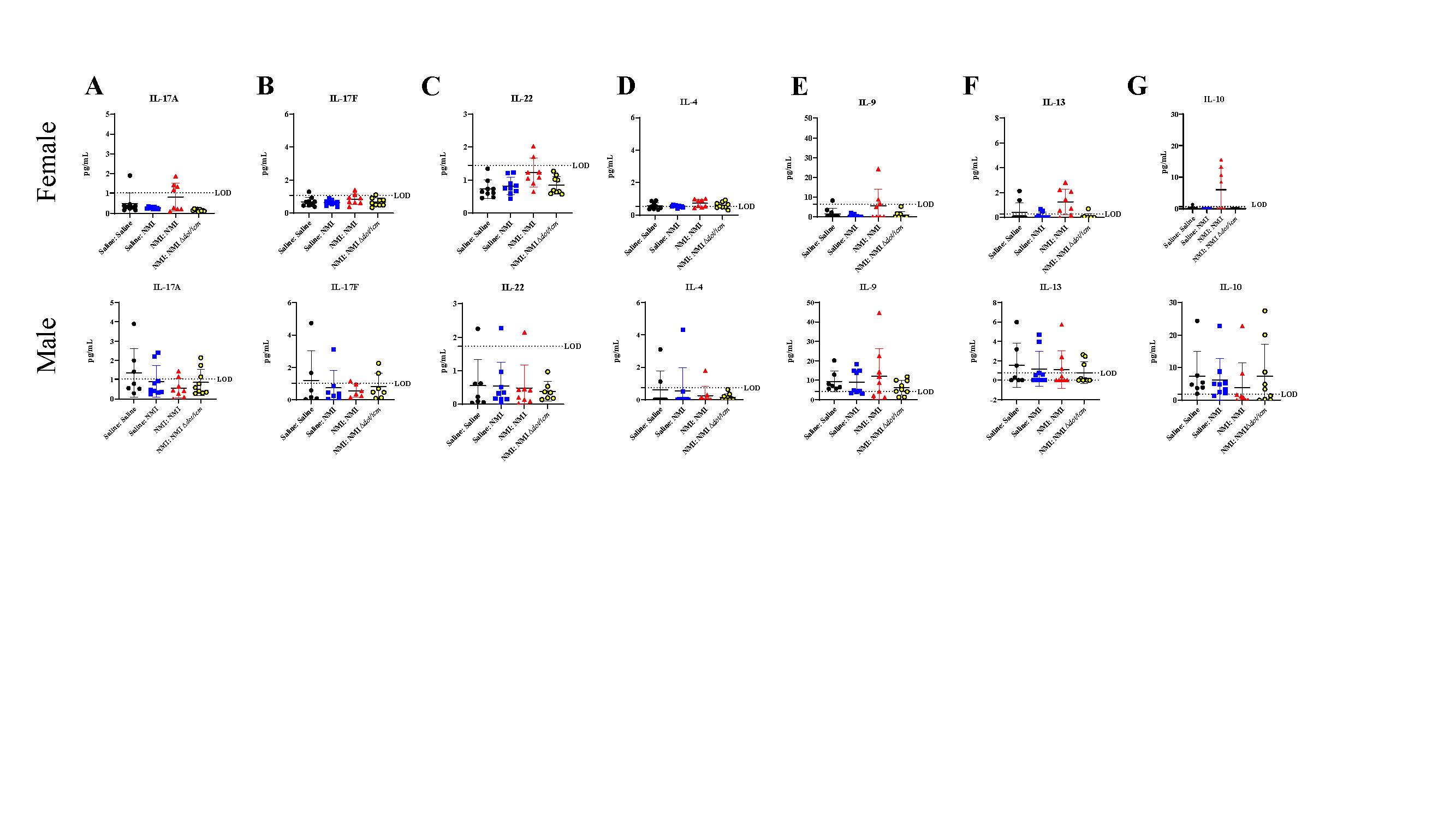

Supplement: Supplementary file 8 [file Image_7.jpeg]

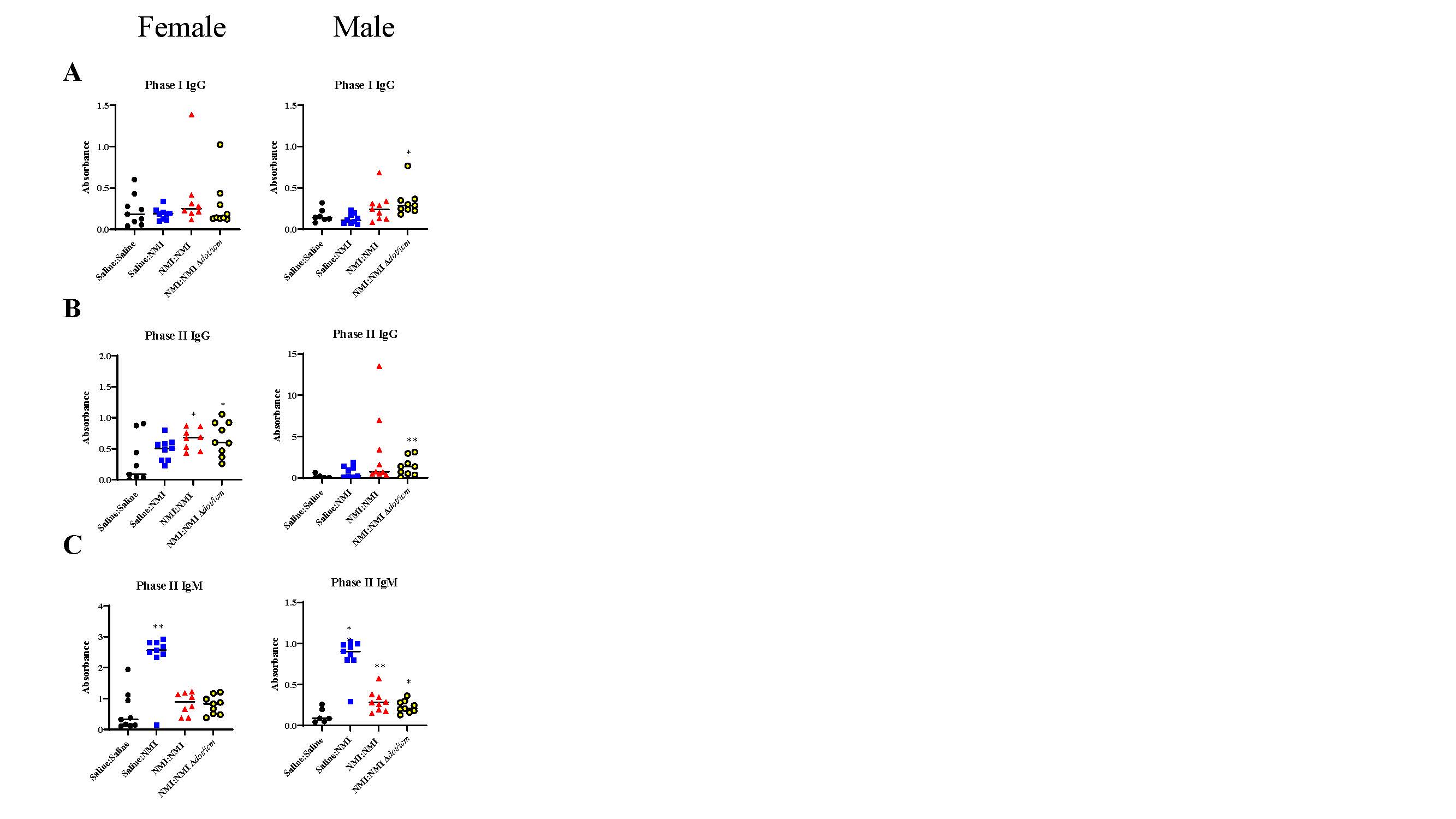

Supplement: Supplementary file 9 [file Image_8.jpeg]

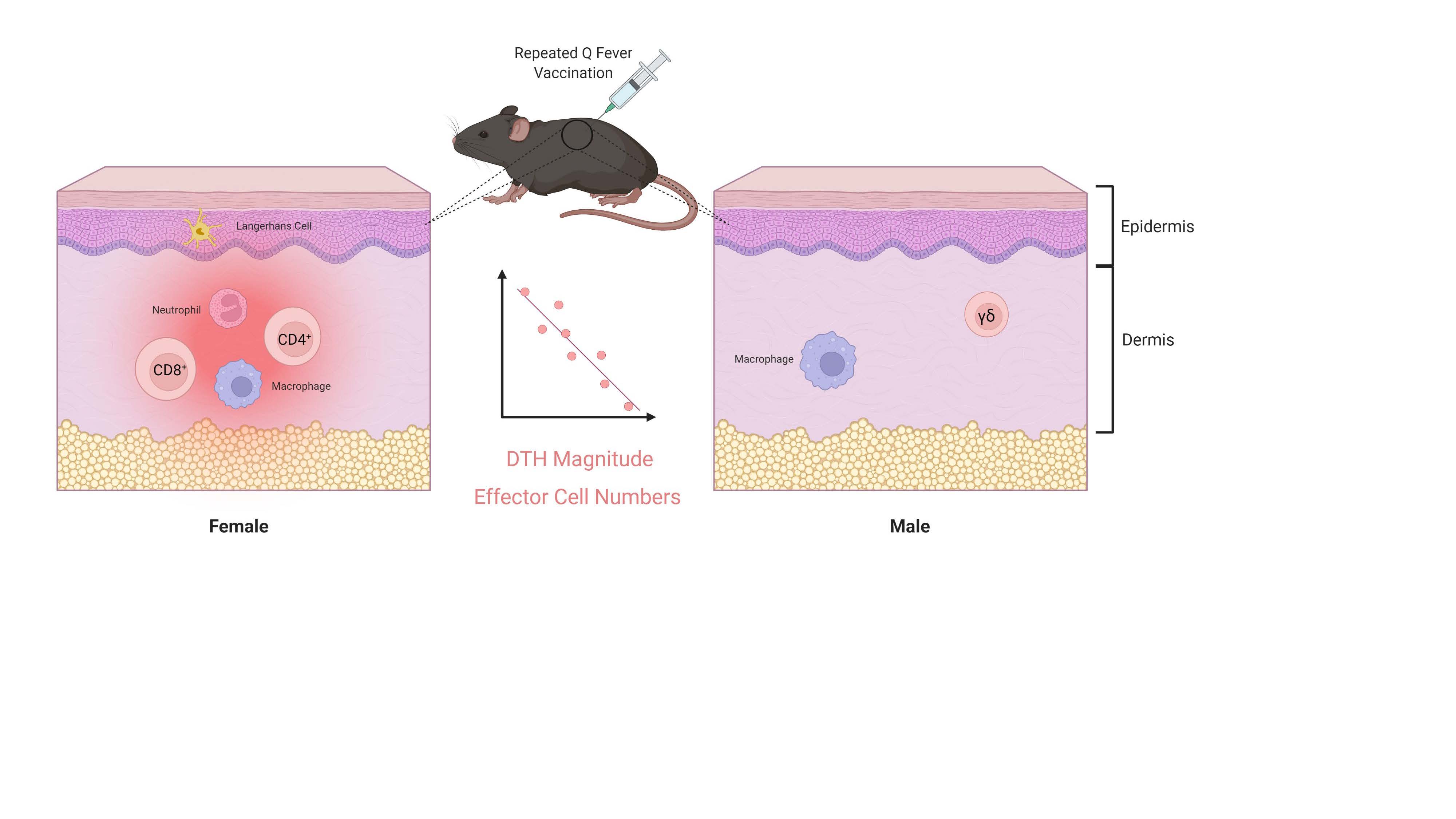

Supplement: Supplementary file 10 [file Image_9.jpeg]
